# Heart Enhancers: Development and Disease Control at a Distance

**DOI:** 10.3389/fgene.2021.642975

**Published:** 2021-03-10

**Authors:** Xuefei Yuan, Ian C. Scott, Michael D. Wilson

**Affiliations:** ^1^Program in Genetics and Genome Biology, The Hospital for Sick Children, Toronto, ON, Canada; ^2^Program in Developmental and Stem Cell Biology, The Hospital for Sick Children, Toronto, ON, Canada; ^3^Department of Molecular Genetics, University of Toronto, Toronto, ON, Canada

**Keywords:** gene regulation, cardiac gene expression, transcription factor (TF), epigenomics and epigenetics, comparative genomics, enhancer

## Abstract

Bound by lineage-determining transcription factors and signaling effectors, enhancers play essential roles in controlling spatiotemporal gene expression profiles during development, homeostasis and disease. Recent synergistic advances in functional genomic technologies, combined with the developmental biology toolbox, have resulted in unprecedented genome-wide annotation of heart enhancers and their target genes. Starting with early studies of vertebrate heart enhancers and ending with state-of-the-art genome-wide enhancer discovery and testing, we will review how studying heart enhancers in metazoan species has helped inform our understanding of cardiac development and disease.

## Introduction

The heart is a vital organ whose primary role is to pump blood through the circulatory system to reach different organs. Heart-like structures are ancient and observed across diverse metazoans, including arthropods (such as *Drosophila*), mollusks (such as octopus) and chordates. Heart structures vary widely across metazoans ranging from a single-layered tubular heart in arthropods and tunicates (including *Ciona*), three separate hearts in some cephalopods (including octopus), a two-chambered heart in jawed fish, a three-chambered heart in amphibians, to a four-chambered heart in other tetrapods (reviewed in Stephenson et al., 2017; Poelmann and Gittenberger-de Groot, 2019). This lineage-specific tuning of cardiac structures is accompanied by changes in the whole circulatory system and highly adapted to the specific physiological needs of different animals. Despite these differences in heart structure, which are mostly related to later-stage heart morphogenesis, many cellular events and molecular regulators involved in early heart development are broadly shared across metazoan species.

A core set of cardiac transcription factors (TFs), including NK2 (*Drosophila* homolog: Tinman), MEF2 (*Drosophila* homolog: Mef2), GATA (*Drosophila* homolog: Pannier), TBX (*Drosophila* homolog: Nmr1/2, Doc1/2/3, etc.), and HAND (*Drosophila* homolog: Hand) families, interact with enhancers to control cardiac gene expression and cell fates in *Drosophila*, fish, and tetrapods (reviewed in Olson, 2006; Tolkin and Christiaen, 2012; Waardenberg et al., 2014). Though specific usage of paralogs and dosage sensitivities may vary between different species, these core TFs form the “cardiac regulatory kernel” (Tolkin and Christiaen, 2012; Waardenberg et al., 2014) in metazoans by closely interacting with each other and extracellular signaling cues. The requirement of extracellular signaling pathways in cardiogenesis also shows a high degree of conservation. The core signaling pathways, such as WNT, FGF, NOTCH, and BMP, play essential cardiogenic roles in both *Drosophila* and vertebrates (reviewed in Noseda et al., 2011).

Early vertebrate heart development involves a conserved sequence of cellular events that are seen in most, if not all, classes of vertebrate species (reviewed in Miquerol and Kelly, 2013). These events include: the emergence of specified cardiac progenitors within the anterior lateral plate mesoderm; migration of the cardiac progenitors to the midline to form the linear heart tube; rightward looping and elongation of the primitive heart tube; ballooning of the atrial and ventricular chambers out from the looped tube; and cardiac cushion and valve formation at the atrioventricular canal and outflow tract. This conserved set of events involve the complex interplay of multiple cardiac cell types, including the first heart field progenitors (FHF) that give rise to the linear heart tube and second heart field progenitors (SHF) that provide later addition to both poles of the heart tube (Kelly, 2012). Although cardiomyocytes make up a significant portion of mature hearts, other cell types, such as endocardial cells, smooth muscle cells, and cardiac fibroblasts, are also involved in cardiac development and physiological function (Hu et al., 2018; Honkoop et al., 2019; Tucker et al., 2020).

Understanding the interplay between multiple cardiac TFs and signaling pathways, within and between the cell types involved in cardiogenesis, requires a detailed knowledge of the *cis*-regulatory elements (CREs) that comprise heart enhancers. The regulatory logic encoded within CREs is readily understood by the embryo and is sufficient to organize multiple cardiac TFs and signaling pathways that ultimately result in a fully formed and functioning heart. In contrast, it has taken decades of experimental advances and insights to develop systems and technologies where cardiac CREs can be discovered and tested.

In this review, we discuss the genetic control of heart development and disease from an enhancer-centric perspective. From early gene-centric enhancer dissection in the 1990s to genome-wide characterization of heart enhancers in development and disease today, the discovery of heart enhancers has substantially shaped our understanding of the principles in cardiac gene regulation. We begin with a brief overview of developmental enhancers followed by a discussion of regulatory principles gained from pre-genomics enhancer studies. We then discuss how rapid advances in genome-wide approaches have transformed our knowledge regarding the locations, interactions, temporal dynamics and functions of heart enhancers. Our review will incorporate evolutionary characteristics of heart enhancers and discuss how new methods for dissecting heart enhancer functions promises to improve our understanding of heart development and cardiovascular diseases.

## Enhancer Structure and Function in Development: A Primer

Enhancers are traditionally defined as short non-coding DNA sequences with the ability to drive gene expression regardless of the genomic distance, position, and orientation relative to the cognate genes [i.e., (Blackwood and Kadonaga, 1998) recently reviewed by Field and Adelman, 2020]. Enhancers can influence gene expression over short (hundreds of base pairs, bp) or large (megabases) genomic distances. These distal enhancers form long-range chromatin interactions with their target genes, such as the well-studied ZRS enhancer that is 1 Mb away from its target *Shh* (Lettice et al., 2003). This flexibility allows a single gene to be regulated by multiple enhancers with different spatiotemporal activities, as well as a single enhancer to contribute to the regulation of multiple genes, which was shown in recent genome-wide enhancer interaction maps (Montefiori et al., 2018; Jung et al., 2019). Together this many-to-many relationship sets up a complex regulatory network to achieve the highly diverse tissue-specific expression patterns evident in development.

Spatial-temporal developmental gene expression is achieved through the combinatorial recruitment of a discrete set of TFs to enhancers (for a recent review of how TFs recognize CREs see Zeitlinger, 2020). TFs interact with enhancers through short degenerate DNA sequence motifs. Recent work investigating the regulatory logic of a typical developmental enhancer supports an overarching principle that specific developmental gene expression relies on sub-maximal TF recognition motifs (Farley et al., 2015). Layered on top of TF motif affinity is the motif syntax within an enhancer, where the spacing, orientation, and order of the motifs themselves can impact the ability of the enhancer to drive developmental gene expression (Farley et al., 2016). It is also important to recognize that developmental genes are commonly regulated by additional redundant enhancers and ascertaining the contributions of individual enhancers remains an outstanding challenge for the majority of developmentally expressed genes (Cannavò et al., 2016; Osterwalder et al., 2018).

Some lineage-determining TFs can bind to compact chromatin regions that are largely inaccessible to other factors. These pioneer factors recruit chromatin-remodeling complexes that promote nucleosome eviction, facilitating the subsequent binding of other collaborating TFs and signal effectors (McPherson et al., 1993; Cirillo et al., 2002; reviewed in Zaret, 2020). To impact gene expression, TFs recruit transcriptional cofactors to enhancers. Cofactors can in turn modify chromatin states by catalyzing post-translational histone modifications (e.g., P300/CBP, MLL3/4), initiate chromatin remodeling (e.g., BRG1), bridge the gap between promoters and enhancer-bound transcription machinery (e.g., Mediator), or affect the affinity of TF binding at enhancers (Malik and Roeder, 2010; Siggers et al., 2011; Slattery et al., 2011; Krasnov et al., 2016). Despite these advances (and many others), much remains to be learned about the mechanisms underlying the recruitment of pioneer factors to a small subset of genomic sites and the molecular events that follow.

Enhancer activation in development is accompanied by progressive changes at the chromatin level, which in turn can be used to annotate enhancer states. Repressed enhancers are located in nucleosome dense regions. Certain repressed regions are characterized by the post-translational histone modification H3K27me3 which is deposited by the Polycomb repressive complex 2 (PRC2). The binding of pioneer factors and chromatin-remodeling complexes may switch enhancers to a poised state, in which enhancers share many features with those in an active state. Poised enhancers show features of low nucleosome occupancy, limited TF binding, and post-translational histone modifications H3K4me1 and H3K4me2 without the presence of H3K27ac, a histone mark of active developmental enhancers (Creyghton et al., 2010; Rada-Iglesias et al., 2011; Zentner et al., 2011). These poised developmental enhancers may even retain the repressive mark H3K27me3 (Rada-Iglesias et al., 2011; Zentner et al., 2011). Upon full activation, transcription co-factor P300 and RNA polymerase II are recruited to enhancers, leading to bi-directional transcription of enhancer RNAs and active enhancer regions marked with H3K27ac (reviewed by Calo and Wysocka, 2013; Heinz et al., 2015).

Enhancer activities are influenced by both local chromatin interactions and higher-order chromatin architectures. Eukaryotic genomes are compartmentalized into large self-interacting chromatin domains, termed topologically associated domains (TADs) (Dixon et al., 2012; Rao et al., 2014). TADs largely constrain the chromatin span that enhancers search through and define the regulatory domains within which enhancer-promoter interactions most frequently occur (Long et al., 2016). For example, promoter capture Hi-C experiments have revealed that 60–80% of the detected promoter interactions occur within TADs (Javierre et al., 2016; Choy et al., 2018; Montefiori et al., 2018). Early studies have noticed that TAD boundaries are shared between different cell types and conserved between species (Dixon et al., 2012; Vietri Rudan et al., 2015), however, these two concepts have been revised more recently. An increasing number of studies reported dynamic loss and gain of TADs and changes of TAD sizes during differentiation (Bonev et al., 2017; Bertero et al., 2019; Zhang et al., 2019). While evolutionarily conserved TADs correspond to regions of conserved synteny harboring important developmental genes and enhancers (Harmston et al., 2017), new analyses have questioned the extent to which TAD boundaries themselves correspond to evolutionary breakpoints (Eres et al., 2019; Eres and Gilad, 2020; Torosin et al., 2020). The importance of understanding how TADs relate to gene regulation is underscored by the increasing number of experiments showing that the disruption of TAD boundaries and sub-TAD domains can rewire enhancer-promoter interactions and fundamentally change the regulatory environment (Guo et al., 2015; Lupiáñez et al., 2015; Flavahan et al., 2016; Franke et al., 2016; Liang et al., 2020).

In sum, the precise and robust transcriptional regulation that occurs during development is achieved by the complex interplay between enhancers, TFs, co-factors, and epigenetic modifications, which together are organized under higher orders of chromatin architectures.

## Heart Enhancers: Fundamental Insights, One Cre at a Time

Studies of heart enhancers initiated from targeted searches around cardiac genes. Putative enhancer regions were screened by “promoter bashing,” in which regulatory regions near the TSS are narrowed down via a series of deletions/mutations to produce overlapping DNA segments that are tested in reporter assays (Table 1). One of the best-studied examples is the mouse *Nkx2.5* locus. LacZ reporter assays identified enhancer elements that specifically drove *Nkx2.5* expression in different chambers of the hearts, as well as in thyroid, pharynx, and stomach within a 14 kb window around the TSS, revealing previously unappreciated complex enhancer modules underlying the control of cardiac TFs. Similar complexities were seen at genes encoding other cardiac TFs, such as *Hand2* (heart and pharyngeal specific enhancers) (McFadden et al., 2000; Charité et al., 2001; Iklé et al., 2012), *Mef2c* (anterior heart field and somite specific enhancers) (Wang et al., 2001; Dodou et al., 2004), and *Gata4* (lateral mesoderm, endocardium, and endoderm specific) (Rojas et al., 2005, 2009; Schachterle et al., 2012). Although limited in number and biased toward proximal gene promoter regions, these studies (and many others) have revealed fundamental principles and mechanisms underlying cardiac gene regulation.

**TABLE 1:** Functionally characterized enhancer regions near cardiac genes.

Target genes	Enhancer length	Genomic position	Expression domain	Upstream regulators or function	References
Mouse *Nkx2.5*	14 kb	5′ flanking sequence of TSS	cardiac crescent, ventricles, outflow tract, pharynx, thyroid, stomach	NKX2.5 (negatively regulate this enhancer)	Tanaka et al., 1999
Mouse *Nkx2.5*	4, 3.3 kb	5′ flanking sequence of TSS	outflow tract, basal portion of the right ventricle, pharynx, thyroid		Tanaka et al., 1999
Mouse *Nkx2.5*	6 kb	3′ flanking sequence of TSS	right ventricle		Tanaka et al., 1999
Mouse *Nkx2.5*	8 kb	[−14, −6 kb] of TSS	medial wall and inner trabeculae of ventricles		Tanaka et al., 1999
Mouse *Nkx2.5*	2.1 kb, two separate fragments (513, 686 bp) (AR1)	[−9.4, −7.3 kb] of TSS	endogenous cardiac expression of *Nkx2.5*	GATA4, MEF2C, NFAT, MZF1	Lien et al., 1999; Chen and Cao, 2009; Clark et al., 2013; Doppler et al., 2014
Mouse *Nkx2.5*	505 bp (AR2)	[−3, −2.5 kb] of TSS	anterior cardiac crescent, right ventricle, outflow tract, developing spleen, pharyngeal pouches	GATA, SMAD4, NFAT, ISL1	Searcy et al., 1998; Liberatore et al., 2002; Lien et al., 2002; Takeuchi et al., 2005; Chen and Cao, 2009; Quinodoz et al., 2018
Mouse *Nkx2.5*	2, 1.5 kb	[−10.7, −3.5 kb] of TSS	early heart tube, outflow tract, right ventricle	GATA	Reecy et al., 1999
Mouse *Nkx2.5*	237 bp (G-S)	[−6.2, −5.79 kb] of TSS	cardiac crescent, heart, forebrain	GATA4, SMAD1/4	Brown et al., 2004
Mouse *Nkx2.5*	10 kb (FL)	5′ flanking sequence of TSS	test in cell lines (10T1/2, P19)	GATA4, SMAD1/4, TBX20	Brown et al., 2004; Takeuchi et al., 2005
Mouse *Nkx2.5*	2.6 kb (UH5)	[−16, −14 kb] of TSS (estimated)	heart tube, both atria, left ventricle, foregut		Chi et al., 2005
Mouse *Nkx2.5*	7.3 kb (UH6)	[14, −6 kb] of TSS (estimated)	right ventricle, interventricular septum, atrial ventricular canal		Chi et al., 2005
Chicken *Nkx2.5*	3 kb, 200 bp	[+976 bp, +3.97 kb], [+2.1, +2.3 kb] of TSS	anterior cardiac cresent, outflow tract, right ventricle, pharyngeal arches (test in mouse)	GATA4/5/6, SMAD, YY1	Lee et al., 2004
Mouse *Gata4*	4.4 kb (G2)	[−45.3, −40.9 kb] of TSS	lateral mesoderm	FOXF1, GATA4, BMP4	Rojas et al., 2005
Mouse *Gata4*	1.9 kb (G9)	93 kb upstream of TSS	cardiac crescent, linear heart tube, endocardium	EST factors (ETS1, ERG)	Schachterle et al., 2012
Zebrafish *gata4*	14.8, 12 kb	5′ flanking sequence of TSS	lateral plate mesoderm, both atrium and ventricle		Heicklen-Klein and Evans, 2004
Zebrafish *gata4*	7.8, 5.5 kb	5′ flanking sequence of TSS	ventricle and the bulboventricular valve		Heicklen-Klein and Evans, 2004
Zebrafish *gata4*	3 kb (DR1), 1.3 kb (DR1A)	[−11, −8 kb] of TSS	lateral plate mesoderm, both atrium and ventricle	TBX	Heicklen-Klein and Evans, 2004
Chicken *GATA5*	500 bp	[−5, −4.5 kb] of TSS	cardiac crescent, septum trans-versum and epicardium, ventricle, AV canal (test in mice)		MacNeill et al., 2000
Mouse *Gata6*	6.8, 1.8 kb	[−4.3, +2.5 kb], [−4.3, −2.5 kb] of TSS	cardiac cresent, high expression in outflow tract	NKX2.5	Molkentin et al., 2000
Chicken *GATA6*	1.4 kb	6.2 kb upstream of TSS	cardiac crescent, high expression in the outflow tract (test in mouse)	NKX2.5	Davis et al., 2000
Chicken *GATA6*	10 kb	[−9.2, +0.8 kb] of TSS	cardiac specific (test in mice)		He and Burch, 1997
Chicken *GATA6*	2.3, 1.5 kb	[−1.5, +0.8 kb], [−1.5 kb, 0] of TSS	posterior region of the heart field, atrioventricular conduction system (test in mice)	Retinoic acid	He and Burch, 1997; Davis et al., 2001
Chicken *GATA6*	317, 187, 102, 47 bp	[−1.4, −1.1 kb] of TSS	atrioventricular conduction system (test in mice)	GATA	Adamo et al., 2004
Mouse *Hand2*	1.5 kb	[−4.2, −2.7 kb] of TSS	cardiac crescent, right ventricle, outflow tract	GATA	McFadden et al., 2000
Mouse *Mef2c*	6, 3.9 kb, 449 bp	[+16.3, +22.5 kb] of TSS	anterior (second) heart field	GATA4, ISL1, NKX2.5, TBX20, TBX1 (negative regulator)	Dodou et al., 2004; Takeuchi et al., 2005; Caputo et al., 2015; Pane et al., 2018
Mouse *Hey2*	2.5, 1.6 kb, 649 bp	211 kb upstream of TSS	cardiac crescent, ventricle and outflow tract	TBX20, GATA4	Ihara et al., 2020
Zebrafish *hey2*	626 bp (aCNE21)	24 kb upstream of TSS	distal linear heart tube, ventricle, outflow tract		Gibb et al., 2018; Yuan et al., 2018
Mouse *Tbx1*	200 bp (require another non-cardiac element)	[−12.8, −12.6 kb] of TSS	second heart field, right ventricle, outflow tract, pulmonary trunk, and pulmonary valves	FOX (likely FOXC1 or FOXC2)	Maeda et al., 2006
Human *TBX5*	368 bp (enhancer 2)	380 kb downstream of TSS	both ventricles and atria	Harbor a CHD-associated variant	Smemo et al., 2012
Human *TBX5*	3.5 kb (enhancer 9)	140 kb downstream of TSS	ventricles, interventricular septum, atrioventricular canal		Smemo et al., 2012
Human *TBX5*	5 kb (enhancer 16)	9 kb upstream	ventricles, interventricular septum, atrioventricular canal, and weakly in atria		Smemo et al., 2012
Mouse *Isl1*	2.9 kb	120 kb downstream	embryonic and adult sinoatrial node (SAN)	SAN hypoplasia and sinus arrhythmia in enhancer knockout, contain SNPs associated with heart rate	Galang et al., 2020
Mouse *Fgf8*	900 bp	[−5.4, −4.5 kb] of TSS	outflow tract, pharyngeal arches	TBX1	Hu et al., 2004
Mouse *Fgf10*	1.7 kb	[+44, +46 kb] of TSS	anterior second heart field, pharyngeal mesoderm	TBX1, NKX2.5 (negative), ISL1	Watanabe et al., 2012
Mouse *Srf*	1 kb, 541 bp	3′ UTR sequence	cardiac crescent, heart tube, tail	TBX2 TBX5, TIP60	Barron et al., 2005

### Establishing Molecular Cascades Regulating Heart Development

**FIGURE 1:**
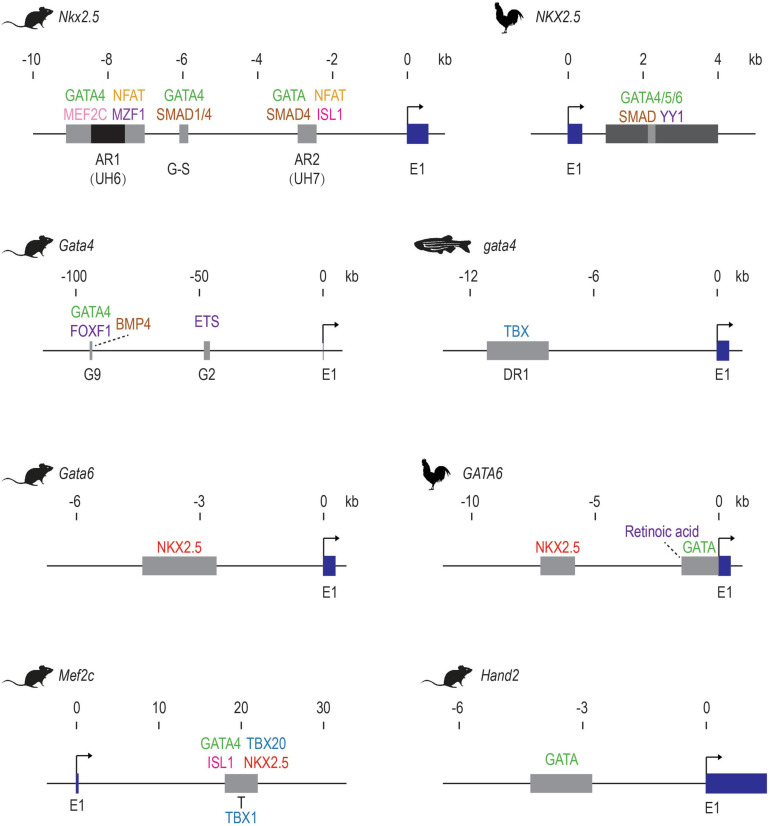
Early examples of validated of cardiac TF-enhancer interactions. The first exons of the cardiac genes are shown in dark blue. Enhancer elements are shown as gray boxes. The AR1 enhancer of mouse *Nkx2.5* contains a repressive element in the middle, which is shown in black. Direct activators are listed above the enhancer elements while repressors are shown below. Upstream factors without direct binding evidence are indicated with dotted lines. E1: exon 1. These schematics are generated based on data from these publications: mouse *Nkx2.5* (Searcy et al., 1998; Lien et al., 1999, 2002; Liberatore et al., 2002; Brown et al., 2004; Chi et al., 2005; Takeuchi et al., 2005; Chen and Cao, 2009; Clark et al., 2013; Doppler et al., 2014; Quinodoz et al., 2018); Chicken *NKX2.5* (Lee et al., 2004); Mouse *Gata4* (Rojas et al., 2005; Schachterle et al., 2012); zebrafish *gata4* (Heicklen-Klein and Evans, 2004); mouse *Gata6* (Molkentin et al., 2000); Chicken *GATA6* (He and Burch, 1997; Davis et al., 2001; Adamo et al., 2004); mouse *Mef2c* (Dodou et al., 2004; Takeuchi et al., 2005; Pane et al., 2018); mouse *Hand2* (McFadden et al., 2000).

Enhancers represent information hubs that integrate multiple upstream regulatory inputs such as lineage-determining master TFs and signaling effectors. Dissecting the transcription factors that bind to enhancers unveils these direct upstream regulators (Figure 1 and Table 1). By combining motif mutagenesis, gel shift, and transgenic assays, *Nkx2.5* enhancer studies revealed that GATA4 and SMAD-mediated BMP signaling directly activated *Nkx2.5* expression through multiple enhancer regions (Searcy et al., 1998; Lien et al., 1999, 2002; Liberatore et al., 2002; Brown et al., 2004) (Figure 1). Dissections of *Nkx2.5* enhancers in the following years added ISL1, TBX20, MEF2C, and NFAT into direct upstream regulators that collectively drove *Nkx2.5* expression in cardiac cells (Takeuchi et al., 2005; Chen and Cao, 2009; Clark et al., 2013). Furthermore, mining known heart enhancers can also lead to discoveries of novel cardiac regulators. For example, MZF1, previously known as a hematopoietic TF, was found to bind to an *Nkx2.5* enhancer from *in silico* motif analysis and validated in embryonic stem cell (ESC) differentiation. Overexpression of MZF1 at different stages of cardiac differentiation revealed its novel, stage-dependent roles in cardiogenesis (Doppler et al., 2014).

Through similar enhancer dissection, the upstream signals of many other cardiac TFs have been identified (Table 1 and Figure 1). For example, the lateral mesoderm expression of mouse *Gata4* relies on transcriptional inputs from FOXF1, BMP4, and its autoregulation (Rojas et al., 2005), while its expression in endocardia requires binding of ETS factors such as ETS1 and ERG (Schachterle et al., 2012). The anterior heart field (AHF) expression of *Mef2c* is positively regulated by GATA4, ISL1, and TBX20 and repressed by TBX1 through an intronic enhancer (Dodou et al., 2004; Takeuchi et al., 2005; Pane et al., 2018). Ventricular expression of *Hey2* is dependent on TBX20 and GATA factor binding, but not NK-2 proteins. Summarizing the existing examples, it is clear that GATA factors, which regulate the expression of many other cardiac TFs (NKX2.5, HAND2, HEY2, MEF2C, etc.), sit among the top of the cardiac molecular cascade. Importantly, sustained cardiac expression of GATA itself requires the transcriptional inputs of other cardiac genes such as NKX2.5 and TBX factors, likely establishing a reciprocal feedback loop to maintain the robustness of the cardiac regulatory network.

### Cardiac TF Crosstalk

Enhancer activation requires the cooperative binding of multiple TFs, therefore studying heart enhancers reveals cooperation and competition between these upstream factors. By co-expressing different combinations of factors together with a specific enhancer, the synergistic effect of factors in activating the enhancer can be revealed by quantitative measures like luciferase assays. Using this type of approach, GATA4 and SMAD1/4 were found to work as mutual co-activators in activating *Nkx2.5* expression through a distal enhancer (commonly referred to as the G-S enhancer) (Brown et al., 2004). At another *Nkx2.5* enhancer (AR1), GATA binding is indispensable for the transcriptional activation mediated by NFAT, likely through cooperative binding (Chen and Cao, 2009). Besides cooperativity, competitive binding between different TFs at heart enhancers can also play an important role in cardiac lineage specification. For example, the two homeodomain TFs, NKX2.5 and ISL1 compete for the same binding sites within an anterior second heart field enhancer of *Fgf10*, reflecting the antagonism between NKX2.5 and ISL1 during the differentiation from SHF progenitors to cardiomyocytes (Watanabe et al., 2012).

### Putting Enhancers to Work

Besides providing direct evidence for building cardiac transcriptional networks, validated cardiac enhancers also frequently serve as genetic tools to label a specific cardiac population of interest for developmental studies. Transgenic mice in which Cre recombinase expression is driven by the *Mef2c* AHF enhancer have been used to determine anterior heart field derived structures and conditionally knock-out many developmental genes (*Mef2c, Tbx1, β-catenin, Ezh2*) to reveal their specific roles in anterior heart field development and congenital heart disease (Verzi et al., 2005; Delgado-Olguín et al., 2012; Barnes et al., 2016; Racedo et al., 2017). A GFP line driven by the *Nkx2.5* AR1 enhancer was used to discover an immature cardiomyoblast population in neonatal mice that was required for normal heart development (Serpooshan et al., 2017). Recently, this *Nkx2.5* enhancer was found to be reactivated after myocardial infarction in the adult heart, suggesting the role of this enhancer in responses to heart injuries (Deutsch et al., 2018). A mouse *Smarcd3* enhancer was found to label early cardiac progenitor cells before the expression of known cardiac markers (*Nkx2.5, Isl1, Tbx5*) in mice, indicating an early molecular distinction between cardiac progenitors and neighboring cells (Devine et al., 2014). This enhancer was later shown to function similarly in zebrafish and helped identify ∼160 putative cardiac enhancers conserved between zebrafish and mammals (Yuan et al., 2018). One of these deeply conserved heart enhancers recapitulated the cardiac expression of the nearby gene *hey2* thus was subsequently used in dissecting how *hey2* restricted cardiac progenitor proliferation (Gibb et al., 2018).

In sum, deeply dissecting cardiac enhancers reveals both molecular tools for visualizing, isolating, and manipulating cardiac populations as well as *cis*- and *trans*-regulatory mechanisms that control cardiac gene expression.

## Unmasking Heart Enhancers With Comparative and Functional Genomics

### Enhancer Hunting: Tools of the Trade

Comparative genomics has long been used to identify putative enhancer regions (Tagle et al., 1988; Aparicio et al., 1995). Such comparative approaches are based on the assumption that functionally relevant enhancer sequences will be under negative selection and will thus show higher sequence constraints than non-functional regions. This assumption is supported by the genome-wide identification of conserved non-coding elements (CNEs) and the following discoveries that many CNEs work as developmental enhancers (Nobrega et al., 2003; Bejerano et al., 2004; Johnson et al., 2004; de la Calle-Mustienes et al., 2005; Shin et al., 2005; Woolfe et al., 2005; Pennacchio et al., 2006). Substantial work using a variety of approaches including transitive alignment (Hiller et al., 2013; Braasch et al., 2016), ancestral reconstruction (Hiller et al., 2013), and conserved microsynteny (Irimia et al., 2012; Clément et al., 2020; Wong et al., 2020) have further enhanced our ability to detect more distantly related conserved non-coding elements.

Although CNEs are enriched for developmental enhancers, the vast majority of enhancers appear to evolve more rapidly, with many being lineage- or species-specific. This feature has been demonstrated in many different tissues or cell types and in both vertebrates and invertebrates (Odom et al., 2007; Kunarso et al., 2010; Schmidt et al., 2010b; Mikkelsen et al., 2010; Cotney et al., 2013; Paris et al., 2013; Arnold et al., 2014; Villar et al., 2015). Although enhancers in different tissues or at different developmental stages may be under varied selection pressures (Blow et al., 2010; Nord et al., 2013; Visel et al., 2013), rapid evolution is an overall feature of enhancer sequences, which suggests that many enhancers would be missed in detection approaches based on sequence conservation alone.

Over the past 15 years, large scale genomic assays have enabled enhancer discoveries at an unprecedented scale (Table 2). In particular, chromatin immunoprecipitation with high-throughput sequencing (ChIP-seq) can locate enhancers by profiling the co-occupancy of lineage-specific TFs, binding of co-factors, or post-transcriptional modifications that marks active enhancers (reviewed in Buecker and Wysocka, 2012). As ChIP-seq requires large numbers of input cells, which is often difficult to obtain from early embryonic tissues, many low input ChIP methods (O’Neill et al., 2006; Brind’Amour et al., 2015) and alternative strategies, such as enzyme-tethering based approaches have been established (e.g., CUT&RUN, CUT&Tag, and CUTAC) (Skene and Henikoff, 2017; Kaya-Okur et al., 2019; Meers et al., 2019; Henikoff et al., 2020).

**TABLE 2:** Genomic approaches for enhancer mapping.

Method category	Method strategy	Description	References
ChIP-seq (detect DNA-binding factor occupancy and histone modification profiles)	Co-factors (EP300, Mediator)	Assays enhancers mediated by specific co-factors; TFs need not be specified in advance.	Blow et al., 2010; He et al., 2011; May et al., 2012
	Co-occupancy of multiple TFs	Reveals specific trans factors but requires specific antibodies for each factor and often each species. Typically requires large numbers of nuclei.	He et al., 2011, 2014; Luna-Zurita et al., 2016; Akerberg et al., 2019
	Active histone marks (H3K27ac, H3K4me1)	Robust antibodies that work across metazoans; reveals enhancer states;requires less input than for TFs.	Wamstad et al., 2012; Nord et al., 2013; He et al., 2014
Enzyme tethering ChIP alternative (use factor-mediated *in-situ* genome fragmentation to profile epigenome)	CUT&RUN (pA-MNase fusion protein)	Unfixed *in-situ* procedure, requires lower cell numbers (∼100 for histone modification) and less sequencing reads	Skene and Henikoff, 2017; Meers et al., 2019
	CUT&Tag (pA-Tn5)	Similar to CUT&RUN with a simpler barcoding step; streamlined workflow in a single tube; works on low cell numbers or even single cells	Kaya-Okur et al., 2019; Henikoff et al., 2020
	CUTAC (pA-Tn5, low salt)	Similar to CUT&Tag with a small modification that detects accessible chromatin in parallel with adjacent histone modifications	Henikoff et al., 2020
Accessible chromatin profiling (detect nucleosome-depleted regions that are enriched for enhancers)	DNase-seq	High quality TF footprintscan be generated.	Thurman et al., 2012; Vierstra et al., 2014, 2020
	ATAC-seq	Simple and robust method that requires low cell numbers, widely applied; can be used on frozen sections; produces a comprehensive list of where CREs may be located.	Buenrostro et al., 2013; Corces et al., 2017
Nascent RNA sequencing run-on assays (depict the real-time activity of RNA polymerases and detect eRNAs)	GRO-seq	Detect actively transcribed eRNAs which is a hallmark of active enhancers	Core et al., 2008
	PRO-seq	Refined version of GRO-seq that uses biotinylated nucleotide to reach nucleotide-resolution, low background, and large dynamic ranges	Kwak et al., 2013; Core et al., 2014
	ChRO-seq	Similar to PRO-seq but use chromatin as starting materials; can be applied to solid tissues and samples with degraded RNAs	Chu et al., 2018
Chromosome conformation capture (use proximity ligation and detect enhancer-promoter interaction)	Hi-C	Maps genome-wide chromatin contacts (‘all-to-all’); requires substantial sequencing to reveal local enhancer-promoter interactions	Lieberman-Aiden et al., 2009
	Promoter capture Hi-C	Maps promoter-centric chromatin interactions; requires less reads for detecting promoter-enhancer interactions	Mifsud et al., 2015; Schoenfelder et al., 2015
	ChIA-PET	Detect chromatin interactions mediated by a specific DNA-binding factor; can enrich rare factor-specific chromatin interactions	Fullwood et al., 2009; Grubert et al., 2020
	HiChIP& PLAC-seq (Use in-situ Hi-C followed by ChIP)	Detects factor-centric chromatin interaction similar to ChIA-PET but require 10-fold to 100-fold fewer cells, also more robust and less time-consuming	Fang et al., 2016; Mumbach et al., 2016
	4C	Identifies all genomic regions that interacts a reference locus (‘one-to-all’); can be used for studying specific enhancers	Simonis et al., 2006

Chromatin accessibility profiling provides a comprehensive view of the candidate regions most likely to harbor CREs, making them arguably the most widely used assay to identify putative enhancers (Thurman et al., 2012; Buenrostro et al., 2013; Vierstra et al., 2014, 2020; Corces et al., 2017). Since active enhancers are transcribed bidirectionally to produce eRNA, nascent RNA sequencing technologies, specifically the run-on assays (GRO-seq, PRO-seq, ChRO-seq, etc.), can be used as a direct readout of enhancer activity. Furthermore, when coupled with chromatin accessibility assays (i.e., ATAC-seq), run-on assays can distinguish active enhancers (producing bi-directional RNAs) from other CREs such as CTCF bound insulators (reviewed in Wissink et al., 2019).

After discovering a distal putative enhancer, one of the most pressing questions is to discover what gene or genes it associates within a cell type and condition of interest. To address this, chromosome conformation capture (3C) based assays (including 4C, 5C, HiChIP, promoter capture Hi-C, and Hi-C) are commonly used to characterize enhancer-promoter interactions (Denker and De Laat, 2016; Fang et al., 2016; Mumbach et al., 2016). Capture Hi-C approaches, such as promoter-capture Hi-C and HiCap (Mifsud et al., 2015; Sahlén et al., 2015; Schoenfelder et al., 2015), are increasingly being used to reveal promoter-centric chromatin interactions at high resolution. Capture-based methods that target putative enhancer regions, such as those discovered by DNAse-seq (Sönmezer et al., 2020), could also be used for ‘enhancer-capture’ Hi-C. Naturally, the choice of 3C-based methods depends on the research question and practical considerations such as the quantity of sample material, genome size, capture probe availability, and sequencing costs.

These widely used genome-scale assays, each with their own strengths (Table 2), continue to reveal new insights into enhancer location, activity and function. The increasing number of high-quality datasets are also creating new opportunities and challenges for integrative data analysis that will further expand our understanding of metazoan heart development and human disease.

### Heart Enhancers: From Genome-Wide Mapping to Metazoan Regulatory Logic

The development of ChIP-chip, ChIP-seq, and other genomic techniques has enabled genome-wide enhancer discoveries and analysis of distinct cardiac samples obtained from diverse model systems (Table 3). Pioneering studies in *Drosophila* using ChIP-chip against master regulators (Twi, Tin, Mef2, Bag, Bin, Doc, and Pnr) and signaling effectors (dTCF and pMad) required for the specification of cardiac mesoderm revealed fundamental principles of combinatorial TF binding dynamics and TF-signaling interactions at cardiac enhancers (Zinzen et al., 2009; Junion et al., 2012). These *Drosophila* cardiac TF mapping studies, together with a comparative analysis of Twi, Tin, Mef2, Bin, and Bap in two distant *Drosophila* species, underscore the conserved presence of combinatorial TF binding, even when the underlying DNA sequence has changed (Khoueiry et al., 2017). The Junion et al. (2012) study led to a “transcription factor collective” model of TF binding where TFs use both protein-DNA and protein–protein interactions to regulate gene expression (reviewed by Spitz and Furlong, 2012), which was later supported by the comparative Khoueiry et al. (2017) study.

**TABLE 3:** Genome-wide metazoan heart enhancer profiling datasets generated using chromatin immunoprecipitation of post translational histone modifications, transcription factors, and cofactors.

Method	Species	Factor	Sample	Condition	Stage	References
BiTS-ChIP-Seq	*Drosophila*	H3, H3K4me3, H3K4me1, H3K27ac, H3K27me3, H3K36me3, H3K79me3	Mesoderm	WT	stages 10–11 (6–8 h AEL, cardiac mesoderm specified)	Bonn et al., 2012
ChIP-seq	Zebrafish	H3.3	myl7:GFP+ cardiomyocytes	Uninjured, 14 days post ablation, 7 days post Nrg1 treatment	Adult	Goldman et al., 2017
ChIP-seq	Zebrafish	H3K27ac	myl7:GFP+ cardiomyocytes	Uninjured, 14 days post ablation	Adult	
ChIP-seq	Mouse	H3K27ac, H3K4me1, H3K4me3, H3K27me3	ESCs, ESC-differentiated cells	WT	ESCs, mesoderm, cardiac precursors, cardiomyocytes	Wamstad et al., 2012
ChIP-seq	Mouse	H3K27ac	Hearts	WT	E11.5, E14.5, E17.5, P0, P7, P21, P56	Nord et al., 2013
ChIP-seq	Mouse	H3K4me1, H3K27me3, H3K4me3	Ventricle	WT	E12.5 and adult	He et al., 2014
ChIP-seq	Mouse	H3K27ac	Ventricle	WT, GATA4 KO	E12.5 (WT, GATA4 KO), adult (normal)	
ChIP-seq	Mouse	H3K27ac	Heart	WT	E12.5	Zhou et al., 2017
ChIP-seq	Mouse	H3K27ac	iCLM (induced cardiac-like myocytes) reprogrammed from MEF	Transfected with GMT, GHMT, AGHMT or mock control	Day 2 and 7 in reprogramming	Hashimoto et al., 2019
ChIP-seq	Mouse	H3K27ac	iCLM (induced cardiac-like myocytes) reprogrammed from MEF	Transfected with single factors	Day 2 in reprogramming	
ChIP-seq	Mouse	H3K27ac	Ventricle, atrium	WT	P4	
ChIP-seq	Human	H3K4me3, H3K27me3, H3K36me3	ESCs, ESC-differentiated cells	WT	pluripotent cells, mesodermal progenitors, specified tripotential cardiovascular progenitors, committed cardiovascular cells, definitive cardiovascular cells	Paige et al., 2012
ChIP-seq	Human	H3K4me3, H3K36me3, H3K27ac, H3K27me3	iPSC-differentiated cells	WT, GATA4_G296S	iPSC-derived cardiomyocytes	Ang et al., 2016
ChIP-seq	Human	H3K27ac, H3K9ac, H3K4me3, H3K4me1, H3K36me3	Left ventricle	Healthy donor and patients with heart failure	fetal, infant, adult (non-failing and failing heart)	Gilsbach et al., 2018
ChIP-seq	Human	H3K27ac	Left ventricle	healthy donors and patients with dilated cardiomyopathy	Adult	Spurrell et al., 2019
ChIP-seq	Human	H3K27ac	ESCs, ESC-differentiated cells	WT	ESCs, mesodermal cells, cardiac mesodermal cells, cardiac progenitors, primitive cardiomyocytes, and ventricular cardiomyocytes	Zhang et al., 2019
ChIP-seq	Human	H3K4me1, H3K4me2, H3K4me3, H3K27ac, H3K27me3, H3K9me3, H3K36me3	Heart	Healthy donor	CS13, CS14, CS16, CS17, CS18, CS19, CS20, CS21, CS23 (Carnegie stage, corresponding to PCW 4–8)	Vanoudenhove et al., 2020
ChIP-chip	*Drosophila*	Twist, Tinman (Nkx2.5)	Whole embryo	WT	Stage 5–7, stage 8–9 (dorsal mesoderm specified), stage 10–11 (cardiac mesoderm specified)	Zinzen et al., 2009
ChIP-chip	*Drosophila*	Mef2	Whole embryo	WT	Stage 5–7, stage 8–9, stage 10–11 stage 12–13, stage 13–15	
ChIP-chip	*Drosophila*	Bagpipe	Whole embryo	WT	Stage 10–11	
ChIP-chip	*Drosophila*	Biniou	Whole embryo	WT	Stage 10–11, stage 12–13, stage 13–15	
ChIP-chip	*Drosophila*	Dorsocross, Pannier, dTCF, and pMad	Whole embryo	WT	Stage 8–9, stage 10–11	Junion et al., 2012
BiTS-ChIP-seq	*Drosophila*	Mef2, Rpb3-Pol II	Mesoderm	WT	stages 10–11	Bonn et al., 2012
ChIP-seq	*Drosophila*	Mef2	Whole embryo	WT		
ChIP-seq	*Drosophila melanogaster* and *Drosophila virilis*	Twist	Whole embryo	WT	Stage 5–7, stage 8–9, stage 10–11	Khoueiry et al., 2017
		Tinman			Stage 8–9, stage 10–11	
		Mef2			Stage 5–7, stage 8–9, stage 10–11 stage 12–13, stage 13–15	
		Bagpipe			Stage 10–11	
		Biniou			Stage 10–11, stage 12–13, stage 13–15	
ChIP-seq	Mouse	P300	Heart	WT	E11.5	Blow et al., 2010
ChIP-seq	Mouse	P300	Heart	WT	P2	May et al., 2012
ChIP-seq	Mouse	GATA4 (flag or biotin-tagged)	Ventricle	WT	E12.5	He et al., 2014
ChIP-seq	Mouse	GATA4 (flag or biotin epitope-tagged)	Ventricle	Normal, banding (surgically placed ligature around the aorta), sham	Adult	
ChIP-seq	Mouse	GATA4, TBX3, NKX2.5, P300	Heart	WT	Adult	van den Boogaard et al., 2012
ChIP-seq	Mouse	HAND2 (flag-tagged)	Limb bud, hearts, branchial arches	WT	E10.5	Osterwalder et al., 2014
ChIP-seq	Mouse	NKX2.5	Heart	WT	E11.5	Dupays et al., 2015
ChIP-exo	Mouse	GATA4, NKX2.5, and TBX5	ESCs, ESC-differentiated cells	WT, NKX2.5 KO, TBX5 KO, double KO	cardiac precursors and cardiomyocytes	Luna-Zurita et al., 2016
ChIP-seq	Mouse	P300 (biotin-tagged)	Heart	WT	E12.5, Adult	Zhou et al., 2017
ChIP-seq	Mouse	P300 (biotin-tagged)	Endocardial and endothelial cells in the heart	WT	Adult	
ChIP-seq	Mouse	CTCF	Left ventricle (isolated cardiomyocytes)	WT, CTCF KO	Adult	Rosa-Garrido et al., 2017
ChIP-seq	Mouse	HAND2 (flag-tagged)	Heart	WT	E10.5	Laurent et al., 2017
ChIP-seq	Mouse	TBX20 (GFP-tagged)	Heart	WT	E11.5	Boogerd et al., 2017
ChIP-seq	Mouse	GATA4, HAND2 (3XTy1 tag), MEF2C (3XTy1 tag), TBX5	iCLM (induced cardiac-like myocytes) reprogrammed from MEF	Transfected with GHMT, AGHMT or single factors	Day 2 in reprogramming	Hashimoto et al., 2019
ChIP-seq	Mouse	GATA4, MEF2C (3XTy1 tag), TBX5	iCLM (induced cardiac-like myocytes) reprogrammed from MEF	Transfected with GMT	Day 2 in reprogramming	
ChIP-seq	Mouse	GATA4, TBX5	Ventricle	WT	P4	
ChIP-seq	Mouse	MEF2A, MEF2C, NKX2.5, SRF, TBX5, TEAD1 (biotin -tagged)	Heart	WT	E12.5	Akerberg et al., 2019
ChIP-seq	Mouse	MEF2A, NKX2.5, SRF, TBX5, TEAD1 (biotin-tagged)	Heart	WT	Adult (P42)	
ChIP-seq	Human	NKX2.5, GATA4, TBX5, SRF, MEF2A, P300 (all TFs biotin-tagged)	HL1 cardiomyocyte cell line	WT	cell line	He et al., 2011
ChIP-seq	Human	P300	Heart	WT	Fetal (gestational week 16), adult	May et al., 2012
ChIP-seq	Human	GATA4, TBX5, MED1	iPSC-differentiated cells	WT, GATA4_G296S	iPS-derived cardiomyocytes	Ang et al., 2016
ChIP-seq	Human	HEY2, NR2F2, and TBX5	iPSC-differentiated cells	WT	cardiomyocytes	Churko et al., 2018
ChIP-seq	Human	CTCF	ESCs, ESC-differentiated cells	WT	ESCs, mesodermal cells, cardiac mesodermal cells, cardiac progenitors, primitive cardiomyocytes, and ventricular cardiomyocytes	Zhang et al., 2019

*Data from consortiums (ENCODE, FANTOM, and Roadmap Epigenomics Projects) are not listed. The table separates post translational histone modifications from TF/cofactor data. For each data type, the experiments are sorted by species first and then by publication date.*

To demarcate the location of putative enhancers active in embryonic and adult hearts, pioneering mammalian studies performed ChIP-seq for the histone acetyltransferase EP300 and the active post-translational histone modification H3K27ac (Blow et al., 2010; May et al., 2012). To overcome the challenge of having to obtain specific antibodies for each TF of interest, many ChIP-seq studies have used tagging methods to biotinylate DNA binding proteins including EP300 and cardiac TFs to define heart enhancers in *Drosophila* (Bonn et al., 2012), mouse embryos (He et al., 2014; Zhou et al., 2017; Akerberg et al., 2019) and human cardiomyocyte cell lines (He et al., 2011). These biotin tagging-based approaches achieve more sensitive and reliable identifications of heart enhancers and enable enhancers discoveries in specific cardiac cell types (Zhou et al., 2017).

Like in *Drosophila*, the combinatorial binding of cardiac TFs defines mammalian heart enhancers (He et al., 2011; Akerberg et al., 2019). However, it is still unclear whether the mammalian cardiac enhancers discovered by these and other studies fit the “TF collective model” proposed for *Drosophila*; the “billboard model (Arnosti and Kulkarni, 2005),” in which specific sets of TFs are recruited to enhancers with flexible motif grammar; or a mixture of models (Long et al., 2016). For example, the importance of heterotypic interactions between mouse TBX5 and NKX2-5 was demonstrated using co-crystal structure together with DNA, as well as ChIP-exo experiments (Luna-Zurita et al., 2016). Intriguingly, the genetic loss of either *Tbx5* or *Nkx2-5* led to ectopic interactions of the other remaining TF. Unlike the more flexible “TF collective” or “billboard” models, TBX5 and NKX2-5 co-occupancy highlighted in this study featured preferred motif arrangements. Most recently, a novel single molecule footprinting (SMF) method was used to ascertain TF co-occupancy in mouse embryonic stem cells (Sönmezer et al., 2020). In this study, simultaneous TF binding did not depend on the identity of the TFs involved, and the co-occupancy of TFs on chromatin lacked of strict motif organization, which the authors proposed agreed with the “billboard model” (Sönmezer et al., 2020). Indeed, comparative approaches using this SMF method to study enhancer logic during metazoan cardiac development will be insightful for both learning general principles governing enhancer regulation as well as the biologically important exceptions that define key physiological processes.

To study cardiac enhancer dynamics across multiple stages of *in vitro* cardiac differentiation or *in vivo* development, several studies from individual labs as well as consortiums, have utilized robust genome-wide assays that do not rely on mapping specific transcription factors, namely ChIP-seq for histone modifications (Paige et al., 2012; Wamstad et al., 2012; Nord et al., 2013; Vanoudenhove et al., 2020), and DNase-seq and ATAC-seq for chromatin accessibility (Bertero et al., 2019; Gorkin et al., 2020; Meuleman et al., 2020). These studies revealed highly dynamic chromatin states accompanying cardiac differentiation and development. Specifically, ATAC-seq is widely used on precious *in vivo* cardiac samples to identify genomic regions that are enriched for TF binding and functional enhancer elements (Jia et al., 2018; Yuan et al., 2018; Pawlak et al., 2019; Racioppi et al., 2019). Recently, accessible chromatin profiling has also enabled the discovery of enhancers specific to cardiac subpopulations, such as pacemaker cells (Galang et al., 2020; van Eif et al., 2020) and endocardial populations (Boogerd et al., 2017).

Functional insights into cardiac enhancer regions continue to be made by studying TF occupancy and chromatin states upon the perturbation of cardiac TFs or signaling pathways in multiple organisms (e.g., *Gata4, gata5*, *Nkx2.5, Tbx5/tbx5, Tbx20, Hand2/hand2, Isl1, Foxf, Fgfr, Mek, and Ras*) (He et al., 2014; Luna-Zurita et al., 2016; Boogerd et al., 2017; Jia et al., 2018; Pawlak et al., 2019; Racioppi et al., 2019), as well as in a human congenital heart disease (CHD) model (cardiomyocytes with a disease-associated missense mutation of *GATA4*) (Ang et al., 2016). These studies reveal the master regulatory roles of cardiac TFs at the chromatin level. For example, GATA4 is essential for establishing open chromatin, promoting active epigenetic modification (H3K27ac) and recruiting TBX5 to the proper cardiac enhancers (He et al., 2014; Ang et al., 2016). On the other hand, TBX5 and NKX2.5 are important for preventing ectopic binding of GATA4 during cardiac differentiation, highlighting the importance of interdependent co-occupancy of these cardiac TFs in precisely controlling cardiac gene expression (Luna-Zurita et al., 2016). This interdependent co-occupancy is also essential in cardiac reprogramming, as only co-expression of cardiac factor cocktails (GATA4, HAND2, TBX5, MEF2C, etc.), but not single-TF overexpression, can leads to robust cardiac TF occupancy to reprogramming enhancers (Hashimoto et al., 2019).

### Heart Enhancers in Space: Chromatin Interactions and Architectures

Heart enhancer activity not only requires proper TF binding, but is under the control of local chromatin interactions and higher-order chromatin architectures. Several groups have conducted promoter capture Hi-C in ESC/iPSC-derived cardiomyocytes or adult hearts to map enhancer-promoter interactions (Choy et al., 2018; Montefiori et al., 2018; Jung et al., 2019). These promoter capture Hi-C studies identified potential target genes for a substantial fraction of candidate heart enhancers. Interestingly, on average 25–35 distal interacting regions per gene and 40–60% of distal regions interacting with more than one gene. Hi-C has also been recently used to profile high-order chromatin architectures such as TADs and compartments across closely sampled time points during the differentiation from stem cells to cardiomyocytes (Bertero et al., 2019; Zhang et al., 2019). These studies showed extensive rearrangement of chromatin architectures during cardiac cell differentiation, with 19% genome switching compartments and 20–40% of TADs being stage-specific. Integrated analyses based on these datasets also revealed important regulatory mechanisms and unknown regulators in heart development. For example, Bertero et al. (2019) detected spatial coalescence of multiple cardiac genes from different chromosomes. This coalescence formed a *trans*-interacting chromatin domain that recruited the muscle-specific splicing factor RBM20 for efficient pre-mRNA splicing (Bertero et al., 2019).

The importance of chromatin interactions and architecture in heart development and function is also revealed by the essentiality of genome organizing factors such as CTCF and the cohesin complex. CTCF knock-out in cardiac progenitor cells leads to severe defects in cardiac cell maturation due to the disruption of enhancer-promoter interaction and subsequent misregulation of cardiac genes (Gomez-Velazquez et al., 2017). In the adult heart, CTCF depletion is sufficient to induce pathological consequences that are very similar to heart failure (Rosa-Garrido et al., 2017). Knock-out of *Stag2* (which encodes a cohesin subunit) in embryonic mice leads to lethality by E10.5 due to severe morphogenesis defects in SHF-derived structures (right ventricle, outflow tract and septation), however, loss of *Stag2* in adults only moderately reduces their fitness, indicating a strong developmental role of Stag2 (De Koninck et al., 2020). Perturbation of the cohesin loading factor NIPBL in both mouse and zebrafish results in multi-organ defects (including heart abnormalities) reminiscent to the Cornelia de Lange Syndrome, a congenital disease linked to *NIPBL* mutation (Kawauchi et al., 2009; Muto et al., 2011; Santos et al., 2016). The different phenotypes observed upon the loss of cohesin complex members, cohesin associated loading proteins, and CTCF indicate that in addition to their roles in sister chromatid cohesion and chromatin organization (Merkenschlager and Nora, 2016; Hanssen et al., 2017; Pugacheva et al., 2020), there are likely more subtle and CTCF-independent roles (i.e., Schmidt et al., 2010a) for these proteins in cardiac gene regulation.

### Enhancing Enhancers With Enhancer-Associated RNAs

Upon activation, many enhancers are transcribed into non-coding RNAs, which are broadly referred to as enhancer RNAs (eRNAs). The expression of eRNAs is well correlated with their putative target gene expression (Kim et al., 2010; Kaikkonen et al., 2013; Li et al., 2013; Andersson et al., 2014; Arner et al., 2015). eRNAs may not only serve as hallmarks of enhancer activation, but also exert important functions in driving target gene expression by promoting chromatin accessibility (Mousavi et al., 2013), mediating enhancer-promoter interaction (Lai et al., 2013; Li et al., 2013; Hsieh et al., 2014), regulating chromatin remodeling (Kaikkonen et al., 2013), and facilitating PolII pause-release at promoters (Schaukowitch et al., 2014; Shii et al., 2017). However, for the vast majority of eRNAs, it remains unclear whether they are simply by-products of enhancer transcription or whether they possess functional roles based on the transcriptional process itself, or through additional molecular interactions in *cis* or in *trans* (reviewed in Li et al., 2016; Arnold et al., 2020).

Though early discoveries described eRNAs as short, non-polyadenylated, bidirectionally transcribed RNAs (Kim et al., 2010; Andersson et al., 2014), a diverse group of molecules with other structures (long, polyadenylated, or unidirectionally transcribed) have been attributed to eRNAs (Koch et al., 2011; Kaikkonen et al., 2013; Li et al., 2013; Alvarez-Dominguez et al., 2017). The structure and functional similarities between some eRNAs and *cis*-acting long non-coding RNAs (lncRNAs) have raised an emerging concept that they represent overlapping categories of regulatory non-coding RNAs (ncRNAs) (Espinosa, 2016; Paralkar et al., 2016; Arnold et al., 2020; Gil and Ulitsky, 2020).

The roles of eRNAs and lncRNAs in the contexts of heart development have been explored by many studies (Grote et al., 2013; Klattenhoff et al., 2013; Ounzain et al., 2014, 2015; Anderson et al., 2016; Alexanian et al., 2017; Yang et al., 2017; Turton et al., 2019; Nicole Ritter et al., 2019). Given the challenge in categorizing these ncRNAs, we consider all ncRNAs that are associated with heart enhancers and discuss the different ways through which they may regulate heart development using two examples: (1) *The ncRNA transcript itself is involved in target gene regulation.* For example, using anti-sense mediated RNA knockdown, Yang et al. (2017) showed that the expression of *Ryr2*, a TBX5 target that is critical for maintaining cardiac rhythm, depends on a novel TBX5-dependent eRNA, *RACER*; and (2) *Instead of the ncRNA molecule, it is the transcriptional activity of the ncRNA locus that appears to be important for controlling the target genes.* Two such ncRNAs come from the *Hand2* locus, upperhand (*Uph*) (Anderson et al., 2016) and handsdown (*Hdn*) (Nicole Ritter et al., 2019). Particularly, the *Hdn* locus interacts with the *Hand2* promoter and putative cardiac enhancers, suggesting it may regulate *Hand2* expression via direct chromatin interaction, reminiscent of CREs (Nicole Ritter et al., 2019). The transcription of *Uph* over a cardiac enhancer upstream of *Hand2* allows the binding of GATA4 and deposition of H3K27ac to this enhancer (Anderson et al., 2016). Together, these examples showcase a few models of the complex interactions between enhancers and the ncRNAs associated with them.

The field of enhancer-associated ncRNAs in heart development has many unanswered questions. Future studies that use chromatin run-on assays (GRO-seq, PRO-seq) or generate deeply sequenced RNA-seq datasets coupled with enhancer annotations should help to understand the dynamic changes of eRNAs in development. Functional experiments such as those use RNA targeting Cas protein (Cas13) (Abudayyeh et al., 2017), shRNA (Lambeth and Smith, 2013), or antisense oligonucleotide-mediated knockdown (Dias and Stein, 2002) will also be essential for teasing out the roles of enhancer-associated ncRNAs in gene-regulation independent of the enhancer elements themselves.

### Heart Enhancers: Keeping Track of Time

As the activity of enhancers are not only tissue-specific but also stage-specific, it is important to obtain high-resolution temporal profiles of heart enhancers to truly understand their function. This is specifically highlighted by the *in vitro* cardiac differentiation study from Wamstad et al. (2012), which showed that enhancers active in ESC, mesoderm progenitors, cardiac progenitors, and cardiomyocytes were largely non-overlapping (Wamstad et al., 2012). Consistently, Luna-Zurita et al. (2016) discovered thousands of GATA4, NKX2.5, and TBX5 binding sites were specific to either cardiac progenitor cells or cardiomyocytes. Similar results have also been reported for *in vivo* development, for example, 80% of the GATA4 binding sites in fetal heart are not occupied by GATA4 in adult heart (He et al., 2014).

Since the heart is the first organ formed in embryogenesis, the embryonic stage that is required to capture the initial phase of cardiogenesis is especially early in development, and is likely during early gastrulation (Scott, 2012; Devine et al., 2014; Lescroart et al., 2014). Though heart enhancers have been extensively characterized across many developmental stages in various species [such as Nord et al. (2013) and Vanoudenhove et al. (2020) and many others in Tables 3–5], there is a paucity of datasets that characterize enhancers active at the initial stage of vertebrate heart development, such as the transition from mesoderm progenitors to cardiac lineages. The majority of *in vivo* studies in vertebrates used relatively mature cardiac samples, including embryonic hearts with defined chamber structures (e.g., E10.5 and onward in mice) or postnatal heart tissues (Tables 3–5). As these stages are later than when cardiac lineage commitment occurs, these studies may not capture the enhancers that specifically drive early cardiogenesis.

**TABLE 4:** Chromatin interaction datasets used for annotating heart enhancers.

Methods	Species	Sample	Condition	Stage	References
Hi-C	Mouse	Left ventricle (isolated cardiomyocytes)	Control, Transverse Aortic Constriction, CTCF KO	Adult	Rosa-Garrido et al., 2017
Hi-C	Human	ESCs, ESC-differentiated cells	WT	ESC-derived mesendoderm cells	Dixon et al., 2015
Hi-C	Human	Left ventricle	WT	Adult	Leung et al., 2015
Hi-C	Human	Right ventricle	WT	Adult	Schmitt et al., 2016
PCHi-C	Human	iPSCs, iPSC-differentiated cells	WT	iPSC, iPSC-derived cardiomyocytes	Montefiori et al., 2018
PCHi-C	Human	ESCs, ESC-differentiated cells	WT	ESC-derived cardiomyocytes	Choy et al., 2018
PCHi-C	Human	Left ventricle	WT	adult	Jung et al., 2019
Hi-C	Human	ESCs, ESC-differentiated cells, iPSCs, iPSC-differentiated cells	WT	ESCs, iPSCs, mesoderm, cardiac progenitors, cardiomyocytes, fetal heart	Bertero et al., 2019
Hi-C	Human	ESCs, ESC-differentiated cells	WT	ESCs, mesodermal cells, cardiac mesodermal cells, cardiac progenitors, primitive cardiomyocytes, and ventricular cardiomyocytes	Zhang et al., 2019

*Data from large consortiums (ENCODE, FANTOM, and Roadmap Epigenomics Projects) are not listed. Datasets are sorted by species first and then by publication dates.*

**TABLE 5:** Chromatin accessibility datasets used for annotating heart enhancers.

Methods	Species	Samples	Condition	Stage	References
ATAC-seq	*Ciona*	B7.5 lineage	WT	6 hpf (native mesoderm),18 hpf (committed heart and pharyngeal muscle precursors)	Racioppi et al., 2019
ATAC-seq	*Ciona*	B7.5 lineage	WT, Fgfr dominant-negative, Mek constitutively active, Foxf-CRISPR, M-Ras constitutively active	10 hpf (multipotent cardiopharyngeal progenitors)	
ATAC-seq	Zebrafish	myl7:GFP+ cardiomyocytes	WT, gata5 -/-, hand2 -/-, tbx5 -/- mutants	72 hpf	Pawlak et al., 2019
ATAC-seq	Mouse	Heart	WT	E12.5	Zhou et al., 2017
ATAC-seq	Mouse	Heart	WT	P1, P14, P56	Quaife-Ryan et al., 2017
ATAC-seq	Mouse	endocardial cells	WT and TBX20 KO	E12.5	Boogerd et al., 2017
ATAC-seq	Mouse	Nkx2-5+ cardiac progenitor cells	WT	E7.5, E8.5, E9.5	Jia et al., 2018
ATAC-seq	Mouse	Isl1+ cardiac progenitor cells	WT and Isl1 KO	E8.5, E9.5	
ATAC-seq	Mouse	Isl1+/CD31+, Isl1+/CD31- cardiac progenitor cells	WT	E8.5, E9.5	
ATAC-seq	Mouse	Isl1+ cardiac progenitor cells	Nkx2.5 overexpression in Isl1+ cells	E9.5, E12.5	
Single-cell ATAC-seq	Mouse	Isl1+ cardiac progenitor cells	WT	E8.5, E9.5	
Omni-ATAC-seq	Mouse	Heart	WT	Adult	Liu et al., 2019
ATAC-seq	Mouse	Ventricle cardiomyocytes	WT	E12.5	Akerberg et al., 2019
ATAC-seq	Mouse	Cardiac pacemaker cells (PCs), right atrial cardiomyocytes (RACMs)	WT	Neonatal (P0-P2)	Galang et al., 2020
Single-cell ATAC-seq	Mouse	Ventricle	myocardial infarction (MI) or sham surgeries	P1, P8 (3days post surgeries for both)	Wang et al., 2020
ATAC-seq	Human	ESCs, ESC-differentiated cells	WT	ESCs, mid primitive streak, lateral mesoderm, cardiac mesoderm	Loh et al., 2016
ATAC-seq	Human	iPSC-differentiated cells	WT, GATA4_G296S	iPS-derived cardiac progenitor cells	Ang et al., 2016
ATAC-seq	Human	ESCs, ESC-differentiated cells, iPSCs, iPSC-differentiated cells	WT	ESCs and iPSCs, mesoderm, cardiac mesoderm, cardiomyocyte	Liu et al., 2017
ATAC-seq	Human	ESCs, ESC-differentiated cells, iPSCs, iPSC-differentiated cells	Control and INN (isotretinoin) treatment	ESCs and iPSCs, mesoderm, cardiac mesoderm	Liu et al., 2018
ATAC-seq	Human	ESCs, ESC-differentiated cells	WT	ESCs, mesoderm, cardiac progenitors, cardiomyocytes	Bertero et al., 2019
ATAC-seq	Human	ESC-differentiated sinoatrial node-like pacemaker cells (SANLPC), ventricle-like cardiomyocytes (VLCM),	WT	ESC-differentiated cardiomyocytes	van Eif et al., 2020

*Data from large consortiums (ENCODE, FANTOM, and Roadmap Epigenomics Projects) are not listed. Datasets are sorted by species first and then by publication dates.*

A few recent *in vivo* studies confirm the observations made from *in vitro* differentiation that enhancer-associated chromatin states are highly dynamic, especially during early cardiac lineage specification. A recent study that profiled mouse *Nkx2.5+* cardiac progenitor cells revealed major changes in chromatin accessibility between E7.5 and E8.5 but only minor differences between E8.5 and E9.5 (Jia et al., 2018). This suggests that early lineage fate transitions may be accompanied by major changes of chromatin states, which become more stabilized in committed cell types. Similar trends are observed in cardiopharyngeal lineage specification in the tunicate *Ciona*, in which most significant chromatin changes occur between the transition from mesoderm progenitors to cardiopharyngeal progenitors compared to later stages (Racioppi et al., 2019). These examples reveal intriguing dynamics of the enhancers involved in early cardiac lineage decisions, however, much remains to be explored. Filling this knowledge gap, especially in the context of developing embryos, can bring valuable insights into key cellular events in early cardiogenesis.

### Evolutionary Mysteries of Heart Enhancers

Intriguing results have emerged from evolutionary studies of heart enhancers. Although the TFs controlling heart enhancers are highly conserved, validated heart enhancers show weak DNA constraint compared to brain enhancers identified at the same developmental stage (E11.5) (Blow et al., 2010). For instance, only 6% of the candidate heart enhancers were deemed to possess high DNA constraint (phastCon score > 600) compared to 44% of forebrain, 39% of midbrain, and 30% of limb enhancers. This could be in part due to the fact that molecularly, the brain seems to be a more conserved organ in terms of the low proportion of positively select genes, old phylogenetic ages of the transcriptomes, and the low percentage of genes showing trajectory changes between different species (Cardoso-Moreira et al., 2019).

It remains an open and intriguing question how heart enhancers that lack evolutionary conservation work together with many conserved cardiac TFs to orchestrate the development of the heart. Several reasons may contribute to this phenomenon. First, it has been demonstrated by many studies that enhancers are rapidly evolving with pervasive turnovers of TF binding sites (TFBSs) (Kunarso et al., 2010; Mikkelsen et al., 2010; Schmidt et al., 2010b; Cotney et al., 2013; Paris et al., 2013; Arnold et al., 2014; Ballester et al., 2014; Villar et al., 2015; Khoueiry et al., 2017). The rapid changes in the sequence, orientation, spacing and numbers of TFBSs within enhancers may not necessarily alter the functional roles of enhancers but do make it hard to detect enhancer sequence homology via genomic sequence alignment. As a consequence, some functionally conserved enhancers will not share detectable sequence homology. A recent and striking example is a sponge *Islet* enhancer, which drives expression that overlaps endogenous *islet* gene (*isl2a*) expression in zebrafish, despite the absence of homologous sequence in the vertebrate genomes. Nevertheless, enhancers with similar TFBS compositions can be found in human and mouse *ISLET/Islet* regions and their activities resemble that of the sponge enhancer in zebrafish (Wong et al., 2020). A similar strategy based on motif composition also identified conserved brain enhancers between chordates and hemichordates, which would not have been detected by sequence alignment alone (Yao et al., 2016). These two examples and many others i.e. (Fisher et al., 2006; Hare et al., 2008; Friedli et al., 2010; Chatterjee et al., 2011) indicate that a grammar more flexible than strict sequence conservation is used in some enhancers to produce conserved transcriptional “output.” Overall, the discordance between sequence and functional conservation may account for a significant portion of the weakly conserved heart enhancers.

**FIGURE 2:**
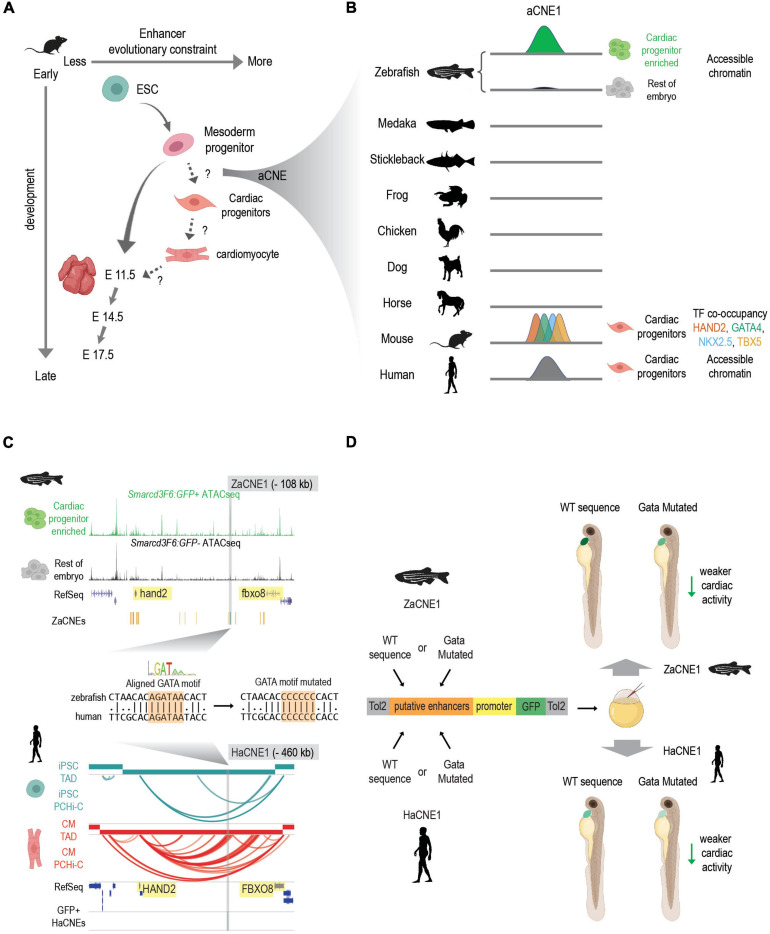
Discovering conserved heart enhancers during early heart development: a case study. **(A)** Enhancers that are active at different stages of heart development show different evolutionary constraints. In mouse, enhancers that are active in mesoderm progenitors show higher sequence conservation than enhancers active in ESC and E11.5 embryonic hearts. But conservation levels of enhancers that active during the transition of mesoderm progenitors to cardiac progenitors and cardiac progenitors to cardiomyocytes remain less characterized. aCNEs, the accessible chromatin shared between zebrafish and human (or zebrafish and mouse) were identified within the mesoderm to cardiac progenitor transition (Yuan et al., 2018). Schematics generated based on Figure 5 (Nord et al., 2013). **(B)** Schematics showing sequence homology and shared enhancer signatures for aCNE1 locus across multiple species. aCNE1 was first discovered as an accessible chromatin region specific for an early cardiac progenitor-enriched population in zebrafish. Gray lines indicate the existence of orthologous sequences to aCNE1 in the given species (based on CNEs identified in Hiller et al., 2013). In mouse, aCNE1 regions are co-occupied by multiple cardiac TFs in cardiac cells (based on data from Luna-Zurita et al., 2016; Laurent et al., 2017). Human aCNE1 region shows chromatin accessibility in cardiac progenitor cells (based on data from Paige et al., 2012). The stickleback and the frog icons were created by Milton Tan and Soledad Miranda-Rottmann, respectively, and shared through (http://phylopic.org/) under the following license (https://creativecommons.org/licenses/by/3.0/). **(C)** Genome browser view of aCNE1 in zebrafish (ZaCNE1) and human (HaCNE1) genome. aCNE1 is located 108 kb upstream of hand2 in the zebrafish genome and 406 kb upstream of HAND2 in the human genome. Yellow boxes highlight the genes flanking aCNE1, indicating the conserved synteny that aCNE1 resides in. ATAC-seq data from Yuan et al. (2018) is plotted for ZaCNE1 and promoter capture Hi-C data from Montefiori et al. (2018) is plotted for HaCNE1. Note that aCNE1 display conserved cardiac-specific activity in both zebrafish (accessibility) and human (interacting with cardiac gene *HAND2*). ZaCNE1 and HaCNE1 shares an aligned GATA motif, the mutation of which can be used to determine if the activity of aCNE1 depends on this GATA motif. **(D)** Functional enhancer assays of WT and GATA motif mutated zebrafish and human aCNE1 sequence in zebrafish embryos. Candidate sequences are cloned into an enhancer vector to drive GFP expression. The whole cassette will be chromatinized after injecting into zebrafish embryos. For both ZaCNE1 and HaCNE1, GATA motif mutation leads to decreased enhancer activity compared to the respective WT sequences. This example illustrates that human and zebrafish aCNE1 share conserved activity and regulation despite less than 60% sequence identity. Schematics generated based on data from Yuan et al. (2018). Parts of this figure were created with BioRender.com.

Second, an increasing number of studies indicate that the conservation of enhancers active in early embryonic development follows an hour-glass like pattern (Bogdanovic et al., 2012; Bogdanović et al., 2016; Liu et al., 2020) similar to that of transcriptomes (Irie and Sehara-Fujisawa, 2007; Domazet-Lošo and Tautz, 2010; Kalinka et al., 2010; Irie and Kuratani, 2011; Yanai et al., 2011). However, much less is known about “phylotypic enhancers” that presumably are established prior to organogenesis to set up conserved vertebrate gene expression patterns. A temporal study of developmental enhancers compared the H3K27ac (a mark of active enhancers) profiles across the development of three mouse tissues (heart, brain, and liver) from ESC to adults (Nord et al., 2013). They showed that both sequence constraints (PhastCon scores) and evolutionary ages of candidate active enhancers peak at different developmental stages in different tissues. Though enhancers active in the brain show the highest conservation at E11.5, heart enhancers active at mouse E11.5 are less conserved compared to those active during earlier cardiac lineage specification (Figure 2A). This suggests that although enhancer turnover is a typical property of heart enhancers, deeply conserved CREs are more likely to be active in early cardiogenesis or even prior to cardiac lineage commitment.

To explore the existence of pre-cardiac enhancers that could contribute to the initiation of cardiac gene regulatory networks, we recently characterized the open chromatin landscape of a cardiac-enriched population in zebrafish embryos before the expression of the canonical cardiac marker *nkx2.5* (Yuan et al., 2018). This approach allowed us to detect cardiac CREs that were primed early in development prior to cardiac lineage commitment. We present this work in Figure 2 as a general example of how comparative genomic resources in combination with epigenomic profiling in two or more species can give insight into functionally conserved developmental enhancers. To determine to what extent deeply conserved CREs were involved in early heart development we exploited conserved non-coding element (CNE) datasets established using both direct alignment and indirect approaches (Hiller et al., 2013; Braasch et al., 2016) and found more than 160 human-zebrafish conserved candidate heart enhancers (referred to as aCNEs). Though most of these aCNEs remain to be tested *in vivo*, the majority of the aCNEs tested (15/18) drive robust cardiac expression in zebrafish. This example illustrates a comparative strategy for discovering early heart enhancers underscores that at least some of the regulatory logic driving vertebrate heart development can be found in orthologous sequences shared between humans and fish.

In sum, despite the overall rapid evolution of heart enhancers, a small fraction of deeply conserved heart enhancers likely contributes to the regulation of early cardiogenesis. The lack of overt sequence conservation in heart enhancers may be partially due to the rapid turnover of TFBSs. On the other hand, variants in heart enhancers that alter gene expression are likely to contribute to morphological differences of cardiac structures between species.

### Heart Enhancers: One Cell at a Time

Currently, most of the data for annotating heart enhancers was generated at the bulk population level (Tables 3–5); however, both *in vitro* differentiated cardiac cells and animal hearts contain heterogeneous populations (reviewed in Paik et al., 2020). This was largely due to the challenges in isolating closely related developmental lineages and collecting enough material from early embryos for enhancer profiling. But as enhancer activity is highly context-specific, the existing data bias likely limits the discoveries of enhancers that are active only in specific subpopulations (e.g., SHF progenitors, endocardial cells, cardiac smooth muscle cells, etc.) or at certain stages.

Rapid advances in single-cell genomics techniques have brought unprecedented opportunities to circumvent the difficulties in cell type isolation. Specifically, single-cell ATAC-seq (scATAC-seq) has become more and more commonly used in delineating cell-type-specific CREs within diverse cellular populations (Buenrostro et al., 2015; Cusanovich et al., 2015). scATAC-seq of *Isl1+* cells from E8.5 and E9.5 mouse embryonic hearts revealed the TF regulators involved in the different stages of two distinct developmental trajectories, the cardiomyocyte and endothelial trajectories (Jia et al., 2018). More recently, scATAC-seq of neonatal hearts post-injury uncovered previously uncharacterized TFs that potentially regulate specific cell types in mammalian heart regeneration and decoded the *cis* and *trans* regulators underlying regenerative and non-regenerative injury responses (Wang et al., 2020). Moreover, large single-cell atlases of chromatin accessibility have been generated for 13 adult mouse organs (∼100,000 nuclei) and 15 fetal human tissues (∼800,000 nuclei), illustrating the regulatory programs that define the cell repertoire for many mammalian organs including the heart (Cusanovich et al., 2018a; Domcke et al., 2020). Embryonic single-cell accessible chromatin landscapes have been profiled for E8.25 mouse embryos (∼19,000 nuclei) and *Drosophila* embryos (∼20,000 nuclei) spanning early blastoderm to terminally differentiated lineages (Cusanovich et al., 2018b; Pijuan-Sala et al., 2020). As all the above studies provide a variety of processed data and interactive web sessions for convenient exploration of the chromatin accessibility of one’s favorite genes or loci, they can be very useful resources for exploring cell type-specific cardiac enhancers.

Furthermore, with single-cell multimodal omics being selected as the Methods of the Year 2019 (Nature Methods, 2020), techniques for simultaneous measuring multiple modalities in the same single cells are blooming rapidly. Related to epigenomics, it has become possible to simultaneous profile accessible chromatin and transcriptome (Cao et al., 2018; Chen et al., 2019; Li et al., 2019; Moudgil et al., 2020), methylome and transcriptome (Angermueller et al., 2016), methylome and chromatin conformation (Li et al., 2019), or even three modalities altogether (Pott, 2017; Clark et al., 2018) within the same cells. The combinatorial use of single-cell epigenomics techniques on cardiac samples will potentially provide a holistic view of enhancer activities in all subtypes of cardiac cells across all stages in heart development. The multi-omics measurements not only enable a more comprehensive and accurate delineation of the state of the single cells but also provide unique opportunities in identifying the potential causal factors across multiple regulatory layers, by correlating changes from genetic, epigenetic, or chromatin conformation levels to the gene expression differences. Although technology and analytic challenges still lie ahead, the application of single-cell epigenomics, especially the multi-omics approaches, into heart development, will likely transform the way that we study and understand heart enhancers and cardiac gene regulatory networks.

### Computing Heart Enhancers

With the rapid accumulation of hundreds of epigenomic and transcriptomic datasets from cardiac tissues, efforts have been made toward compiling them and extract sequence features from known cardiac enhancers to predict unknown ones. Dickel et al. (2016) conducted an integrative analysis of over 35 genome-wide H3K27ac or P300 profiles from mouse or human heart samples to compile a compendium of more than 80,000 heart enhancers, which serves as one of the most comprehensive putative heart enhancer lists available to date. The abundance of genomics datasets and the growing number of *in vivo* validated heart enhancers also provide ample input for building computational models for novel heart enhancer prediction. One kind of model is purely based on the sequence features of the gold standard heart enhancers experimentally validated *in vivo*. For example, Narlikar et al. (2010) combined motif discovery, Markov sequence feature characterization, and linear regression to build a heart enhancer classifier from ∼70 validated heart enhancers. They used this classifier to discover more than 40,000 putative heart enhancers within the conserved CNEs in the human genome, with an *in vivo* validation rate > 60% (Narlikar et al., 2010). By comparing validated cardiac and non-cardiac enhancer sequences from *Drosophila*, Jin et al. (2013) identified a novel motif as a classifier for heart enhancer prediction. They further showed that this motif was essential for driving cardiac activity in 3/8 enhancers tested. One widely used sequence-based machine learning method, gapped k-mer support-vector-machine (gkm-SVM) (Ghandi et al., 2014), has been applied to learn the sequence features from previously identified open chromatin regions. It predicted an addition of 80,000 putative cardiac CREs and the cognate TFs that bind to them (Lee et al., 2018).

Several studies have explored how including different genomics features in training models could affect their performance in enhancer prediction. A study in *Drosophila* added ChIP signals on top of sequence motifs into their classifiers and found this combined strategy significantly boosted the prediction accuracy of cell-type-specific cardiac enhancers than motif sequence alone (Ahmad et al., 2014). By further including ChIP data for a larger set of cardiac TFs and histone modifications, their updated model was able to distinguish enhancers active in distinct subpopulations of cardiac cells and pericardial cells in *Drosophila* embryos (Busser et al., 2015). Similarly, Akerberg et al. (2019) took advantage of the variety of ChIP-seq data that they generated for mouse hearts and compared the performance of different chromatin features (open chromatin, H3K27ac histone modification, cardiac TF occupancy) alone or combined in predicting heart enhancers. They found open chromatin had high sensitivity while TF binding profiles yielded high precision in enhancer prediction. Ultimately, the number of co-bound cardiac TFs turned out to be the most important classifier in heart enhancer prediction compared to signal intensities (Akerberg et al., 2019). With the rapid evolvement of the machine learning field, computational classification and predictions will become an important component that is complementary to experimental data in heart enhancer characterization. The two strategies will benefit from the advancement of each other and together expand our understanding of enhancer biology.

## Heart Enhancers in Cardiovascular Disease

Heart diseases are a leading cause of death worldwide (Mozaffarian et al., 2015). As the most prevalent human birth defects, congenital heart disease (CHD) affects roughly 0.8% of newborns (Fahed et al., 2013). Though disruption of a set of developmental and structural genes have been recognized as the causes of a portion of CHD, the genetic factors underlying a large number of cases remain ambiguous (Fahed et al., 2013; Barnett and Postma, 2015; Postma et al., 2015; Richter et al., 2020). Genome-wide association studies (GWAS) have been carried out to identify the underlying genetic causes of a wide range of cardiovascular phenotypes and diseases, including CHD, cardiac arrest, coronary artery disease (CAD), cardiac arrhythmia, cardiomyopathy, and myocardial infarction (Arking et al., 2014; Nikpay et al., 2015; Eppinga et al., 2016; Nelson et al., 2017). Currently, thousands of variants have been implicated in heart-related disease risks (NHGRI GWAS catalog^1^).

Whole-genome sequencing (WGS) is becoming the method of choice for discovering *de novo* variants in CHD. Supporting the use of WGS for discovering molecular mechanisms underlying CHD, a recent study illustrated that the potential contribution from disruptive non-coding variants was at least as high as that from coding-variants (Richter et al., 2020). However, several factors complicate the functional annotation of disease-associated non-coding variants (Zhang and Lupski, 2015). In the case of common genetic variation associated with CHD-related phenotypes uncovered by GWAS, the tagged SNPs used will be in linkage disequilibrium (LD) with other SNPs that may represent the true causal variant. Even if a likely pathogenic non-coding mutation or copy number variation is nominated, one must then ascertain when and where this change impacts development and disease. In the following section, we briefly review insights into heart enhancer function revealed by human genetic studies.

### Connecting Non-coding Variants to Cardiovascular Diseases

Only a handful of non-coding variants linked to cardiovascular diseases have been functionally dissected (Table 6). Compared to studying the function of a protein coding gene mutation, the functional characterization of non-coding disease associated variants is challenging. An early example of this was done for a genetic variant on human chromosome 9p21 harboring multiple SNPs associated with myocardial infarction and CAD (reviewed by Samani and Schunkert, 2008). A large 70 kb deletion of the whole orthologous sequence in the mouse genome severely reduced the expression of the nearby cardiac genes (*Cdkn2a/b*) and affected aortic smooth muscle cell proliferation and senescence. Allele-specific analysis of *Cdkn2b* transcripts in the heterozygous mice revealed a lack of *cis*-acting enhancers as the main mechanism underlying *Cdkn2b* downregulation, suggesting this genetic susceptibility interval contains enhancers that could be affected by the discovered sequence polymorphisms (Visel et al., 2010). However, disruption of *cis*-regulatory elements is not the only mechanism that contributes to diseases risk. Other studies revealed that expression of the long non-coding RNA (lncRNA) *ANRII*, which resides in chromosome 9q21, was affected by several SNPs within this region, and *ANRII*, in turn, could regulate other genes involved in vascular cell proliferation, adhesion, apoptosis, and remodeling (Holdt et al., 2010; Congrains et al., 2012a, b).

**TABLE 6:** Functionally characterized non-coding SNPs implicated in cardiovascular disease.

SNP	SNP position	Gene(s)	Disease	Evidence	References
SNPs within a 58 kb interval, include cis-regulatory elements	chr 9p21	*Cdkn2a/b*	coronary artery disease	Deletion of the mouse orthologous interval severely impairs *Cdkn2a/b* expression nearby through a cis-acting mechanism.	Visel et al., 2010
chr12:114704515: G>T, overlaps a *TBX5* enhancer	90 kb downstream of *TBX5*	*TBX5*	Septal defects	The risk allele ablates the cardiac enhancer activity	Smemo et al., 2012
rs118026695:A>G and g.4574C>deletion	*NKX2.5* promoter	*NKX2.5*	ventricular septal defect	Risk alleles significantly upregulate the promoter activity	Pang et al., 2012
g.17483564C>T and g.17483576C>G	*NKX2.5* enhancer, 10 kb upstream	*NKX2.5*	ventricular septal defect	Conserved with mouse AR1 *Nkx2.5* enhancer, risk alleles significantly decrease the enhancer activity	Huang et al., 2013
rs12190287:C>G rs12524865:C>A overlap enhancers	3′ UTR of *TCF21*	*TCF21*	coronary heart disease	The protective alleles disrupts AP-1 binding and enhancer-associated histone modification, leading to *TCF21* expression changes.	Miller et al., 2013
rs12190287:C>G, overlaps a miRNA binding site	3′ UTR of *TCF21*	*TCF21*	coronary heart disease	The protective allele (G) changes *TCF21* transcript structure and disrupts miR-224 binding and post-transcriptional repression mediated by this miRNA. TGF-b and PDGF-bb signaling act upstream of miR-224 mediated allele-specific expression.	Miller et al., 2014
rs6801957:G>A, overlaps an enhancer	Intron of *SCN10A*	*SCN5A*	cardiac rhythm disorder	The enhancer interacts with the *SCN5A* promoter. The minor allele disrupts a Tbox binding site and impairs the enhancer activity in the cardiac conduction system.	van den Boogaard et al., 2012, 2014
rs7539120:A>T	An upstream enhancer of *NOS1AP*	*NOS1AP*	QT interval variations	The risk allele leads to increased enhancer activity. Overexpression of NOS1AP result in altered electrophysiology in cardiomyocytes	Kapoor et al., 2014
rs4897612:G>T	−137 in *VNN1* promoter	*VNN1*	HDL cholesterol levels	eQTL of *VNN1*, allele-specific transcriptional activity, chromatin accessibility, binding of nuclear protein including SP-1,	Kaskow et al., 2014
rs2050153:G>A	−587 in *VNN1* promoter	*VNN1*	HDL cholesterol levels	eQTL of *VNN1*, allele-specific chromatin accessibility, methylation and chromatin condensation	
rs138912749:T>C overlaps a miRNA binding site	3′ UTR of *SHOX2*	*SHOX2*	atrial fibrillation	The minor allele creates a functional binding site for miR-92b-5p, which leads to reduced expression of *SHOX2*.	Hoffmann et al., 2016
rs6489956:C>T overlaps two miRNA binding sites	3′ UTR of *TBX5*	*TBX5*	CHD susceptibility	The minor allele shows increased binding to miR-9/30a, which leads to reduced expression of *TBX5*	Wang et al., 2017
rs7373779, rs41312411, rs11710077, rs13097780, rs6801957	*SCN5A-SCN10A* GWAS locus	*SCN5A*	QT interval variations	Allele-specific enhancer activity and nuclear factor binding. (More putative variants were identified other than these five representative ones)	Kapoor et al., 2019

Another well-studied example is rs12190287, a CAD-associated variant located within the 3′ UTR of the *TCF21* gene. Two continuous studies together revealed a dual mechanism of this SNP in modulating *TCF21* expression at both transcriptional and post-transcriptional levels (Miller et al., 2013, 2014). Overlapping a *TCF21* enhancer, this variant causes dysregulation of *TCF21* through allele-specific histone modifications (H3K4me1, H3K27ac, H3K27me1) and AP-1 factor (c-Jun, JunD, ATF3) binding. These allele-specific chromatin effects are further augmented upon PDGFR-β stimulation, which indicates that the vascular growth factor signaling also acts differently on this variant (Miller et al., 2013). Moreover, the same minor allele disrupts a miR-224 binding site within the 3′ UTR of *TCF21*, therefore, prevents the post-transcriptional repression of *TCF21* mediated by this miRNA (Miller et al., 2014).

The ion channel genes *SCN5A/SCN10A* locus is another hotspot heavily loaded with variants linked to cardiac arrhythmia and conduction system disorders (Veerman et al., 2015). One cardiac arrhythmia-associated SNP rs6801957 is located within the intron of *SCN10A* but is encompassed by a human-mouse conserved enhancer that interacts with the nearby gene *SCN5A* (van den Boogaard et al., 2014). This variant, but not other variants in LD disrupts the binding of TBX3/TBX5 *in vitro* and reduces the activity of this enhancer in the cardiac conduct system (van den Boogaard et al., 2012). Overall, these variant-oriented studies revealed the molecular mechanisms through which single nucleotide substitutions could alter enhancer activity and lead to pathological gene expression.

### Discovering Disruptive Non-coding Variants Near Cardiac Genes

The CREs controlling the expression of TFs (i.e., the regulators of the regulators) are prime candidate regions for discovering damaging mutations that lead to gene dosage-related phenotypes (van der Lee et al., 2020). Indeed, hypothesis driven dissection of enhancers near cardiac genes have revealed several examples of disease causing non-coding mutations that control haploinsufficient cardiac genes *TBX5*, *NKX2.5*, and *SHOX2* (reviewed in Chung and Rajakumar, 2016; Steimle and Moskowitz, 2017; Li et al., 2018).

It had been known for over a decade that heterozygous mutations within *TBX5* lead to Holt-Oram syndrome in humans (Basson et al., 1997, 1999) when Smemo et al. (2012) went searching for disease-causing enhancer mutations around the *TBX5* gene in families with septal defects, the predominant cardiac defect of Holt-Oram syndrome. This study, which involved scanning more than 700 kb for conserved non-coding sequences revealed three enhancer elements which together recapitulated the endogenous TBX5 heart expression in developing mouse embryos. Targeted sequencing revealed homozygous mutations in one of the enhancer elements in individuals with, but not in family members without, the disease. Another targeted sequencing of the *NKX2.5* locus in ventricular septal defect patients revealed novel variants within the *NKX2.5* promoter and a known distal enhancer (AR1). These novel variants significantly altered the transcriptional activity of the *Nkx2.5* promoter and AR1 enhancer in luciferase assays (Pang et al., 2012; Huang et al., 2013). These tour de force experiments illustrate the lengths one must go to implicate regulatory mutations as a disease causing mechanism, and demonstrates how understanding the molecular mechanisms underlying human disease can reveal fundamental biological insights in cardiac enhancer elements.

In addition to enhancers and promoters, non-coding regulatory variation can impact miRNA binding sites, lncRNAs, or even several of these functional elements at the same time. In principle this could occur by disrupting or creating TF/miRNA binding sites, changing chromatin states, mediating different responses to extracellular signaling, or affecting lncRNA expression which in turn can affect gene regulation in trans (Table 6). For example a variant associated with increased CHD susceptibility was identified within the 3′ UTR of *TBX5*. This variant was shown to increase the binding of two miRNAs with the minor allele leading to a significant reduction in the expression of TBX5 through transcriptional and translational regulation (Wang et al., 2017). *NKX2.5* mutations have also been implicated in diverse types of CHD, including ventricular septal defects (reviewed in Chung and Rajakumar, 2016). Similarly, target sequencing of the *SHOX2* region in atrial fibrillation (AF) patients identified an AF-associated SNP within the 3′ UTR. The 3′ UTR allele created a binding site for an mRNA miR-92b-5p, which significantly reduced the *SHOX2* 3′UTR reporter activity in a luciferase assay (Hoffmann et al., 2016).

While there are relatively few hard-won examples of non-coding mutations that explain the molecular mechanism behind CHD, it is clear that a comprehensive annotation of heart enhancer location and function will accelerate molecular-based diagnoses and our understand of heart gene regulation.

### Interpreting Non-coding Variants With Genome-Wide Enhancer Annotation

With the burst of cardiac epigenomic datasets in the past decade, the interpretation of heart disease-associated variants has developed from susceptible locus-centric to a genome-wide manner. Continuous efforts have been made to first establish a comprehensive enhancer annotation and then use for the fine-mapping non-coding variants (Dickel et al., 2016; Choy et al., 2018; Montefiori et al., 2018). For example, the heart enhancer list that they curated from ChIP-seq datasets, Dickel et al. (2016) found more than 2000 enhancer-overlapping variants that were associated with heart phenotypes. When deleting two of the variant-containing enhancers that were upstream of cardiac structure genes (*Myl7* and *Myl2)*, they showed that both enhancers are required for normal cardiac gene expression, cardiomyocyte morphology, and heart functions. On top of enhancer identification, chromatin conformation capture assays are especially helpful for linking cardiac GWAS SNPs to their targeted genes. The promoter capture Hi-C datasets generated in differentiated cardiomyocytes arguably pinpoint the true target genes of many GWAS and LD SNPs, some of which were different from the target genes proposed based on proximity (Choy et al., 2018; Montefiori et al., 2018). Remarkably, Montefiori et al. (2018) reported that 90% of the SNP-gene interactions skipped at least one gene promoter, arguing against the intuitive approach of assigning SNPs to their neighboring genes when interpreting possible causal mechanisms. In line with the cell-type-specificity of enhancer activities, the interaction networks identified using cardiomyocyte promoter capture Hi-C data turned out to be most informative to interpret cardiac arrhythmia phenotypes (which directly results from cardiomyocyte dysfunction) as compared to CHD, CAD, heart failure, and myocardial infarction (all of which involved cellular systems other than cardiomyocytes) (Choy et al., 2018; Montefiori et al., 2018). This indicates that generating chromatin maps for other cardiac cell types or at other differentiation stages could more effectively facilitate the mechanistic dissection of other types of cardiovascular diseases.

With the promising future of functional genomics in non-coding variants dissection, generation and curation of transcriptome and epigenome datasets have been tailed toward studying a specific type of heart disease to achieve higher precision. For example, to understand causal variants for atrial fibrillation (AF), RNA-seq data and ATAC-seq specifically from the left atria were generated to identify potential CREs and target genes that were likely to be affected by the genetic variants within 104 AF-associated loci (van Ouwerkerk et al., 2019). Following this study, a functional enhancer screening of these AF-associated loci using STARR-seq found 24/55 the variant-containing enhancers with allele-specific activities, demonstrating the robustness of this approach. Deletion of the orthologous region of one such enhancer near *Hcn4* in the mouse genome caused a loss of *Hcn4* expression and cardiac defects (van Ouwerkerk et al., 2020).

In addition to our growing understanding of the regulatory logic underlying developmental gene expression, it is also important to acknowledge the contribution of pro-inflammatory processes on heart enhancer usage and gene expression. For instance, the rapid pro-inflammatory gene expression by the NF-κB transcription factor complex, which across cell types utilizes clusters of strong enhancers (also known as “super enhancers”) to rapidly deploy pro-inflammatory gene expression (Brown et al., 2014; Schmidt et al., 2015). This mode of gene regulation can recruit transcriptional machinery from cell-lineage genes in a process known as cofactor squelching (Schmidt et al., 2015, 2016). Indeed a detailed knowledge of acute and chronic inflammatory enhancer biology during heart development and disease is essential and integrating this information with emerging compendiums of heart epigenomic data (such as Vanoudenhove et al., 2020) will be valuable.

Integrating enhancer information into the functional annotation of non-coding variants is no doubt a powerful approach; however, it should be noted that disrupting enhancer activities is not the only mechanism underlying the pathological consequences of non-coding variants. Even with extensive efforts in curating heart enhancers, nearly 90% of the heart disease-associated LD SNPs did not overlap any heart enhancers in the compendium (Dickel et al., 2016) and more than 80% of them could not be linked to gene promoters based on cardiomyocytes promoter capture Hi-C data (Montefiori et al., 2018). Apart from other possible technical reasons, this small overlap suggests regulatory mechanisms other than altering heart enhancers could account for a substantial portion of non-coding variants-mediated disease risk. In fact, unbiased examination of 98 amplicons (250–600 bp) containing 106 SNPs linked to QT interval phenotypes at the *SCN5A* locus found that 35% of the reference allele-containing amplicons showed enhancer activity while another 44% worked as silencers in luciferase assays (Kapoor et al., 2019), suggesting disease-associated SNPs likely fall into not only enhancers but also silencers. Besides CREs, functional non-coding variants have also been mapped to miRNA-binding sites and lncRNAs (Table 6). A recent CHD genomic analysis has demonstrated significant enrichment of RNA-binding-protein regulatory sites in *de novo* variants identified in CHD patients, indicating contribution from disrupted post-transcriptional regulation to CHD (Richter et al., 2020). Moreover, it has been shown that the same minor allele of a variant could regulate the target gene expression through both transcriptional and post-transcriptional mechanisms and, even more strikingly, in an opposite manner, highlighting the complexity of sequence polymorphisms in affecting gene expression (Miller et al., 2013, 2014). Therefore, a comprehensive annotation of different types of cardiac CREs that are not limited to enhancers, together with a good non-coding RNA annotation, will be necessary for truly understanding the mechanisms of the heart disease from the non-coding variant perspective. Additionally, it is likely that several coding and/or non-coding variants collectively explain a complex cardiovascular phenotype. Thus while it is important to dissect disease phenotype associated variants individually, more complex studies looking at genetic interactions and addictive effects may well be required.

## Emerging Techniques for the Functional Dissection of Heart Enhancers

So far, numerous putative heart enhancers have been identified in different conditions and cell types from several model organisms. However, compared to enhancer mapping, the throughput of current approaches for enhancer functional dissection, especially *in vivo*, remains a major bottleneck. Traditionally, each candidate enhancer is accessed individually via being placed upstream of a reporter gene and introduced into cells or *in vivo* organisms. Collective efforts using this approach have led to the establishment of central resources of validated enhancers, such as the Vista Enhancer Browser^2^ (Visel et al., 2007). To measure enhancer activity in a more high throughput manner, several methods have been developed through the years, such as massively parallel reporter assays (MPRA) (Melnikov et al., 2012; Patwardhan et al., 2012; Sharon et al., 2012), and self-transcribing active regulatory region sequencing (STARR-seq) (Arnold et al., 2013). However, most of these approaches are typically carried out *in vitro* or in the absence of chromatin contexts, raising the question of how faithfully their results reflect the native activities of the candidate regions. Recently, the development of more robust and scalable *in vivo* enhancer assays, such as the site-directed enhancer-reporter assay (enSERT), has allowed systematic assessment of more than 100 variants in an essential limb enhancer (Kvon et al., 2020). For invertebrates like *Drosophila*, unbiased, automated enhancer mutational scanning has been established using robotic systems, which permits multi-stage quantitative measurement of enhancer activities in development (Fuqua et al., 2020). Developing similar systems for vertebrates will greatly improve our capacity in assessing vertebrate enhancer functions and advance our understanding of how regulatory information is encoded in developmental enhancers.

Compared to all enhancer reporter assays, which introduces an atypical distance between candidate enhancers and the reporter genes, a complementary perhaps preferred way to understand enhancer functions is to dissect their activity and function in their endogenous loci. The ever-growing CRISPR-Cas9 toolbox provides many options for *in situ* enhancer dissection (reviewed in Klein et al., 2018; Pickar-Oliver and Gersbach, 2019; Xu and Qi, 2019). Individual enhancer deletions or substitutions have been routinely used to characterize enhancer functions in specific developmental processes (Dickel et al., 2016, 2018; Kvon et al., 2016; Osterwalder et al., 2018; van Eif et al., 2020; van Ouwerkerk et al., 2020). To increase the throughput, a variety of CRISPR-based enhancer screens have been developed for *in vitro* systems, such as the saturated tilling arrays that can unbiased assess certain genomic loci for functional enhancers (Korkmaz et al., 2016; Diao et al., 2017; Gasperini et al., 2017) and epigenetic screens against candidate enhancers using deactivated Cas9 (dCas9) coupled with transcriptional activators or repressors (Klann et al., 2017; Simeonov et al., 2017; Fulco et al., 2019; Gasperini et al., 2019). Specifically, by using single-cell RNA-seq as readouts, CRISPR-mediated epigenetic screens have been successfully applied to perturb thousands of candidate enhancers in cell lines to determine their functional importance and target genes (Fulco et al., 2019; Gasperini et al., 2019). Though achieving the same throughput *in vivo* may still be challenging, increasing efforts have been made toward applying these powerful systems in animals. Very recently, a single-cell-based *in vivo* CRISPR/Cas9 screen (Perturb-seq) has been successfully used to screen 35 genes in the mouse developing neuronal cortex *in utero* (Jin et al., 2020). Though not large-scale yet, this study offers a very encouraging framework to achieve systematic assessment of genes or CREs *in vivo*. Moreover, dCas9-mediated epigenetic perturbation, which is likely more suitable for enhancer screens, has been continuously optimized over the years and showed a promising future of targeting enhancers in a more scalable manner in developing animals (Morita et al., 2016; Zhou et al., 2018; Li et al., 2020).

## Discussion, Concluding Remarks, and Future Perspectives

The past decade has witnessed an exponential growth of the numbers of putative heart enhancer regions identified, largely owing to rapid advances in epigenomic profiling approaches. These techniques are still growing at an ever-increasing speed and will undoubtedly continue to revolutionize the way that researchers annotate and interpret enhancer activities. Single-cell epigenomic techniques, especially the multi-omics approaches, will likely become one of the main driving forces in expanding the horizon of cardiac enhancers and regulatory networks in the next decade. However, it should be noted that many analytical challenges are inherently associated with single-cell epigenomic datasets that currently remain sparse and noisy (reviewed in Schwartzman and Tanay, 2015; Verma and Kumar, 2019). Robust computational and statistical models are needed to extract biological information from other irrelevant signals (e.g., technical noises, batch effect) and for integrating the multimodal data of different characteristics, dimensionalities, and coverages to model them in a single space. Methods addressing these challenges are rapidly emerging (reviewed in Forcato et al., 2020; Hao et al., 2020) but still in the early stages in terms of accommodating all different data types and features. Both technical improvements of assay sensitivity and the development of analytic methods are essential for successfully applying these single-cell genomics techniques to understanding enhancer biology.

*In vivo* functional characterization of enhancers, especially developmental enhancers, is still one of the biggest challenges lying ahead. As developmental genes are usually regulated by multiple enhancers with overlapping activities, it is reasonable to assume that most enhancers may have redundant functions in normal development (Frankel et al., 2010; Perry et al., 2010; Cannavò et al., 2016; Dickel et al., 2018; Osterwalder et al., 2018). While these redundant enhancers may be seemingly dispensable in normal conditions, they could be required in stressed environments or sensitized genetic backgrounds (e.g., such as heterozygous deletion of developmental TFs) (Frankel et al., 2010; Perry et al., 2010; Osterwalder et al., 2018). It therefore becomes a very complicated task to determine the specific contexts in which a given developmental enhancer is required.

On the other hand, we are in an era with unprecedented opportunities to overcome these challenges. The combined use of CRISPR technologies and single-cell genomics is likely to make a substantial contribution to functional enhancer dissections in the near future. With the concurrent advancement of these two technologies, it probably will not be too far until we can conduct mid- to large-scale *in vivo* enhancer screening. Moreover, coupling CRISPR with other single-cell epigenomic assays (e.g., single-cell accessibility chromatin) to target TFs or chromatin modifiers (Rubin et al., 2019; Sanjana et al., 2020), can provide information complementary to enhancer screens and together build toward a comprehensive regulatory network.

From traditional approaches to the newest genomic assays, the rich history of heart enhancer studies has not only left us with a wealth of knowledge about the genomic locations, functional roles, evolutionary conservation, and disease implications of heart enhancers but also opened up many challenges and unanswered questions. What are the best experimental designs and analytic strategies of single-cell epigenomic assays? How can we increase the scalability of functional enhancer assays and efficiently adopt them into *in vivo* contexts? Could we develop more robust and transferable computational methods that can not only predict heart enhancers but also determine their chamber-, cell-type or developmental-stage specific activities and how the activity of enhancers can be affected by non-coding variants? We may not be sure when these questions will be fully answered, but we can confidently anticipate that efforts made in tackling these challenges will push our understanding of heart enhancers and cardiac regulatory network to an unprecedented level.

## Author Contributions

XY researched, conceived the structure, created the figures and tables, and led the writing of the review. MW and IS developed the ideas and provided text for the review. All the authors read and edited the review.

## Conflict of Interest

The authors declare that the research was conducted in the absence of any commercial or financial relationships that could be construed as a potential conflict of interest.

## Footnotes

1 https://www.ebi.ac.uk/gwas/

2 https://enhancer.lbl.gov/
